# Visualization of ER-to-Golgi trafficking of procollagen X

**DOI:** 10.1247/csf.24024

**Published:** 2024-09-06

**Authors:** Yuan Ximin, Hitoshi Hashimoto, Ikuo Wada, Nobuko Hosokawa

**Affiliations:** 1 Laboratory of Molecular and Cellular Biology, Institute for Life and Medical Sciences, Kyoto University, Kyoto 606-8507, Japan; 2 Department of Cell Science, Institute of Biomedical Sciences, Fukushima Medical University, School of Medicine, Fukushima 960-1295, Japan

**Keywords:** collagen, GFP-procollagen X, ER-to-Golgi trafficking, export from ER, TANGO1

## Abstract

Collagen is the most abundant protein in the extracellular matrix of animals, and 28 types of collagen have been reported in humans. We previously analyzed the endoplasmic reticulum (ER)-to-Golgi transport of fibril-forming type III collagen (Hirata *et al.*, 2022) and network-forming type IV collagen (Matsui *et al.*, 2020), both of which have long collagenous triple-helical regions. To understand the ER-to-Golgi trafficking of various types of collagens, we analyzed the transport of short-chain type X collagen in this study. We fused cysteine-free GFP to the N-telopeptide region of procollagen X (GFP-COL10A1), as employed in our previous analysis of procollagens III and IV, and analyzed its transport by live-cell imaging. Procollagen X was transported to the Golgi apparatus via vesicular and tubular carriers containing ERGIC53 and RAB1B, similar to those used for procollagen III. Carriers containing procollagen X probably used the same transport processes as those containing conventional cargoes such as α_1_-antitrypsin. SAR1, TANGO1, SLY1/SCFD1, and BET3/TRAPPC3 were required for trafficking of procollagen X, which are different from the factors required for trafficking of procollagens III (SAR1, TANGO1, and CUL3) and IV (SAR1 and SLY1/SCFD1). These findings reveal that accommodation of various types of collagens with different shapes into carriers may require fine-tuning of the ER-to-Golgi transport machinery.

## Introduction

Collagens are the most abundant protein components of the extracellular matrix in animals. Twenty-eight types of collagen have been reported in humans, including type I, a major component of skin and bone; type II, a building block of cartilage; type III, a component of the vascular wall, and type IV, a constituent of the basement membrane (Kadler *et al.*, 2007; Gordon and Hahn, 2010; Ricard-Blum, 2011; Mouw *et al.*, 2014). In addition to typical fibril-forming collagens and other long-chain collagens, there are several short-chain collagens with relatively short triple-helical collagenous regions (Sutmuller *et al.*, 1997; Mouw *et al.*, 2014). The α chains of procollagens are synthesized in the endoplasmic reticulum (ER), and three α chains form a triple helix via the association of non-collagenous C-propeptides (Myllyharju and Kivirikko, 2004; Canty and Kadler, 2005). The collagen triple-helical regions are comprised of Gly-X-Y repeats, and hydroxylation of Pro at the Y position stabilizes the collagen triple helix. Procollagens undergo additional oligosaccharide modifications in the Golgi apparatus and are then secreted.

Extensive analyses have been performed to elucidate how large collagen molecules exit the ER and traffic to the Golgi apparatus. Fibrillar collagens and network-forming type IV collagen are 300–400 nm long, which exceeds the diameter (60–80 nm) of normal coat protein complex II (COPII) vesicles (Miller and Schekman, 2013; Malhotra and Erlmann, 2015). Thus, several transport mechanisms have been proposed, including mega carriers that accommodate long and rigid collagen molecules (Nogueira *et al.*, 2014; Santos *et al.*, 2015; Gorur *et al.*, 2017), saccules lacking COPII coats that are discharged from protruding ER exit sites (ERES) where procollagen is packed in a COPII-dependent manner (Mironov *et al.*, 2003), and a short-loop pathway that does not use vesicles (McCaughey *et al.*, 2018 reviewed by Malhotra and Erlmann, 2015; McCaughey and Stephens, 2019; Raote and Malhotra, 2019). By performing live-cell imaging, we recently reported that GFP-procollagen III is transported from the ER to the Golgi apparatus via vesicular and tubular carriers containing ERGIC53 and RAB1B (Hirata *et al.*, 2022), while GFP-procollagen IV moves to the Golgi via unique transport vesicles without recruitment of ER-Golgi intermediate compartment (ERGIC) membranes (Matsui *et al.*, 2020). Another study reported that fluorescently-tagged procollagen I is transported out of the ER by vesicles based on live-cell imaging (Omari *et al.*, 2020).

To analyze ER-to-Golgi transport of short-chain procollagens, we constructed fluorescently-tagged procollagen X by introducing cysteine-free SGFP (Suzuki *et al.*, 2012) into the N-telopeptide region so that the tag would not interfere with trimer formation in the ER or hamper lattice formation in the extracellular matrix. Type X collagen is a network-forming short-helix collagen produced by hypertrophic chondrocytes of cartilage (Schmid and Linsenmayer, 1985; Schmid *et al.*, 1990; Eyre, 1991). Expression of type X collagen is restricted to the hypertrophic zone of the growth plate and the calcified zone of articular cartilage of long bones. It consists of a homotrimer of α chains encoded by the *COL10A1* gene and contains a triple-helical region of 463 amino acids with flanking N-terminal NC2 (38 amino acids) and C-terminal NC1 (161 amino acids) domains (Ninomiya *et al.*, 1986). The type X collagen molecule is 100–130 nm long (Schmid *et al.*, 1984; Kwan *et al.*, 1991; Frischholz *et al.*, 1998), and mutations of the *COL10A1* gene cause Schmid metaphyseal chondrodysplasia (Lachman *et al.*, 1988; Bateman *et al.*, 2005; Wilson *et al.*, 2005). We next searched for proteins required for procollagen X secretion. To this end, we used p52 cells (Wagner *et al.*, 2000), which are derived from HEK293 cells and stably express procollagen X as well as the α and β subunits of proryl-4-hydroxylase. Co-expression of proryl-4-hydroxylase (Myllyharju, 2008) helps to synthesize procollagen X containing appropriate hydroxyproline and increases its stability (Wagner *et al.*, 2000; Shoulders and Raines, 2009).

In the present study, we revealed that GFP-COL10A1 was transported from ERES via tubulo-vesicular carriers containing ERGIC membranes. The diameter of the trafficking vesicles was 400–550 nm, which is similar to that of carriers containing procollagens III and IV. SAR1, SLY1/SCFD1, TANGO1, and BET3/TRAPPC3 were necessary for secretion of procollagen X, which differ from the proteins required for trafficking of procollagens III and IV (Matsui *et al.*, 2020; Hirata *et al.*, 2022).

## Materials and Methods

### Plasmid construction

The human *COL10A1* gene was cloned from p52 cells, which were kindly provided by Dr. von der Mark (Max-Planck Institute of Biochemistry, Germany), by RT-PCR and subcloned into pcDNA3.1(+). To construct GFP-COL10A1, cysteine-free SGFP2 (Suzuki *et al.*, 2012) containing the GST-linker at the N-terminus was introduced into the endogenous XhoI site located in the telopeptide of COL10A1. mScarlet-RAB1B and mCherry-α_1_-antitrypsin (α1AT) were constructed as described previously (Hirata *et al.*, 2022). PDI-mCherry, Golgi-BFP, mCherry-ERGIC53, and GFP-α1AT were described elsewhere (Matsui *et al.*, 2020). The SAR1[H79G] mutant containing EYFP at the N-terminus was used (Kamada *et al.*, 2004). To produce lentivirus vectors, PSPAX2, pCMV-VSV-G-RSV-Rev, and pCSII-CMV-MCS were obtained from the RIKEN Bioresource Research Center (Tsukuba, Japan). TANGO1S was cloned into pMH (Roche Diagnostics, Basel, Switzerland) by RT-PCR using total RNA extracted from HEK293 cells to introduce a HA tag. TANGO1L-HA was kindly provided by Dr. Malhotra (CRG, Spain) and Dr. Saito (Akita University, Japan). The coding regions of TANGO1S-HA and α1AT were subcloned into the AfeI site of pCSII-CMV-MCS. As a control, a tandem SGFP2 (Kremers *et al.*, 2007) dimer-expressing vector, pCSII-tdSGFP2, or pCSII-tagRFP (Evrogen, Moscow, Russia) was used.

### Small interfering (siRNA) oligonucleotides

siRNA oligonucleotides targeting TANGO1, BET3, SLY1/SCFD1, STX18, and ZW10 were described previously (Hirata *et al.*, 2022). The sequences used to target CUL3 were described elsewhere (Matsui *et al.*, 2020). Medium GC negative control siRNA (Invitrogen, Waltham, MA, USA) was used as control siRNA.

### Antibodies

A mouse monoclonal antibody against human procollagen X (X53) was kindly provided by Dr. von der Mark (Girkontaite *et al.*, 1996). A rabbit anti-GFP antibody was generated as described previously (Sakurai *et al.*, 2018). Other antibodies were purchased from the following suppliers: mouse anti-MIA3/TANGO1 (Santa Cruz Biotechnology, Dallas, TX, USA), mouse anti-CUL3 (Santa Cruz Biotechnology), rabbit anti-SLY1/SCFD1 (CUSABIO Technology, Wuhan, China), mouse anti-STX18 (Santa Cruz Biotechnology), mouse anti-ZW10 (Santa Cruz Biotechnology), rabbit anti-TRAPPC3/BET3 (CUSABIO Technology), mouse anti-actin (Millipore, Billerica, MA, USA), rabbit anti-calnexin (Enzo Life Sciences, Farmingdale, NY, USA), rabbit anti-SEC23 (Affinity BioReagents, Golden, CO, USA), rabbit anti-calreticulin (CRT, Affinity BioReagents), horseradish peroxidase-conjugated anti-rabbit IgG (BTI; Thermo Fisher Scientific, Rockford, IL, USA), horseradish peroxidase-conjugated anti-mouse IgG (Zymed Laboratories/Thermo Fisher Scientific), Alexa Fluor 594-conjugated goat anti-rabbit IgG (Thermo Fisher Scientific), and Alexa Fluor 488-conjugated goat anti-mouse IgG (Thermo Fisher Scientific).

### Cell culture, transfection, and drug treatment

The HT-1080 human sarcoma cell line (ATCC, CCL-121) and p52 cells (Wagner *et al.*, 2000) were kindly provided by Dr. Klaus Kuhn and Dr. von der Mark, respectively (Max-Planck Institute of Biochemistry). Lenti-X^TM^ cells (TaKaRa, Otsu, Japan) were used to produce lentiviruses. Cells were grown in Dulbecco’s modified Eagle’s medium supplemented with 10% fetal bovine serum. Lentivirus vectors were produced according to a previously described method (Miyoshi *et al.*, 1998), except PEI Max^TM^ (Polysciences, Inc., Warrington, PA, USA) was used for transfection. A Lenti-X Concentrator^TM^ (Clontech/TaKaRa, Otsu, Japan) was used to concentrate lentiviruses. TransIT LT1 (TaKaRa) and RNAi-MAX (Invitrogen) were used to transfect plasmids and siRNA, respectively. To induce ER-to-Golgi transport of procollagen X and GFP-COL10A1, ascorbic acid phosphate (WAKO, Osaka, Japan) was added to the medium at a final concentration of 136 μg/mL. Cycloheximide (CHX; Nacalai Tesque, Kyoto, Japan) was dissolved in phosphate-buffered saline and added to the medium at a final concentration of 100 μM.

### TANGO1L- and TANGO1S-knockout (KO) HeLa cells

TANGO1L- and TANGO1S-KO HeLa cells were established by transfecting each sgRNA vector, pSpCas9(TANGO1L) or pSpCas9(TANGO1S), and cloning cells lacking expression of TANGO1L or TANGO1S. The targeting sequences of TANGO1L and TANGO1S were 5'-gccttctatcgccgctaccc-3' and 5'-cagcactggccggcggttct-3', respectively. pSpCas9 was obtained from Addgene (pX458).

To transduce TANGO1S-HA into TANGO1S-KO HeLa cells, cells were plated on 3.5 mm dishes, and freshly prepared lentivirus expressing TANGO1S-HA was added the next day after concentration using a Lenti-X Concentrator^TM^. Cells were passaged 24 h after lentivirus transduction, and GFP-COL10A1 was transfected using TransitLT1. Cells were cultured for a further 2 days before harvesting. pCSII-RFP-expressing lentivirus was transduced as a control. Wild-type (WT), TANGO1L-KO, and TANGO1S-KO HeLa cells transduced with lentivirus encoding α1AT were passaged the next day and harvested after incubation for an additional 2 days.

### Live-cell imaging

Live-cell imaging was performed as described previously (Matsui *et al.*, 2020; Hirata *et al.*, 2022). Briefly, cells were grown on 35-mm glass-bottom dishes and transfected with the indicated plasmids. Images were acquired using a Leica TCS SP8 confocal microscope (Leica Microsystems, Wetzler, Germany) equipped with a 63×/1.4 N.A. oil immersion objective, and were analyzed with LAS AF (Leica). The temperature was set to 37°C with 5% CO_2_ using a microscope incubator (Tokai Hit Co., Ltd., Shizuoka, Japan). To measure vesicle sizes, images (1024 × 1024 pixels) were acquired, and the diameters were measured manually. For photo-bleaching, a 100% laser beam was focused on the intended area.

### Immunocytochemistry

Immunocytochemistry was performed as described previously (Matsui *et al.*, 2020; Hirata *et al.*, 2022). For cell staining without permeabilization, cells were processed without Triton X-100 treatment.

### Western blotting

Cells were lysed in cell lysis buffer (50 mM Tris-HCl (pH 7.6), 150 mM NaCl, and 5 mM EDTA) containing 1% NP-40 and protease inhibitors (0.2 mM AEBSF, 2 mM NEM, 1 μg/mL leupeptin, and 1 μg/mL pepstatin). To detect procollagen X and GFP-COL10A1, 100% TCA and cell lysis buffer lacking NP-40 were added to the cell lysate to final concentrations of 10% TCA and 0.1% NP-40, respectively (Frischholz *et al.*, 1998). Culture medium (serum-free medium (SFM)) was centrifuged at 500 × g for 5 min, and the supernatant was collected. Thereafter, 100% TCA and cell lysis buffer containing 1% NP-40 were added to final concentrations of 10% TCA and 0.1% NP-40. Both the cell lysate and culture medium were kept on ice for 10 min and centrifuged at 13,000 × g for 15 min. After removing the supernatant, the pellet was washed with ice-cold acetone, air-dried, dissolved in 1× Laemmli’s buffer containing 0.1 M DTT, separated by SDS-PAGE, and blotted onto PVDF membranes (Millipore). Immunoblotting and analysis were performed as described previously (Matsui *et al.*, 2020; Hirata *et al.*, 2022).

### Statistical analysis

All results are indicated by means ± standard deviation (s.d.). The numbers of replicates are described in the figure legends. Statistical analysis was performed using the two-tailed Student’s *t*-test and Dunnett’s test. *P*<0.05 was considered statistically significant. *P<0.05; **P<0.01; ***P<0.001; and n.s., not significant.

## Results

### Synthesis and secretion of GFP-COL10A1

**Fig. 1:**
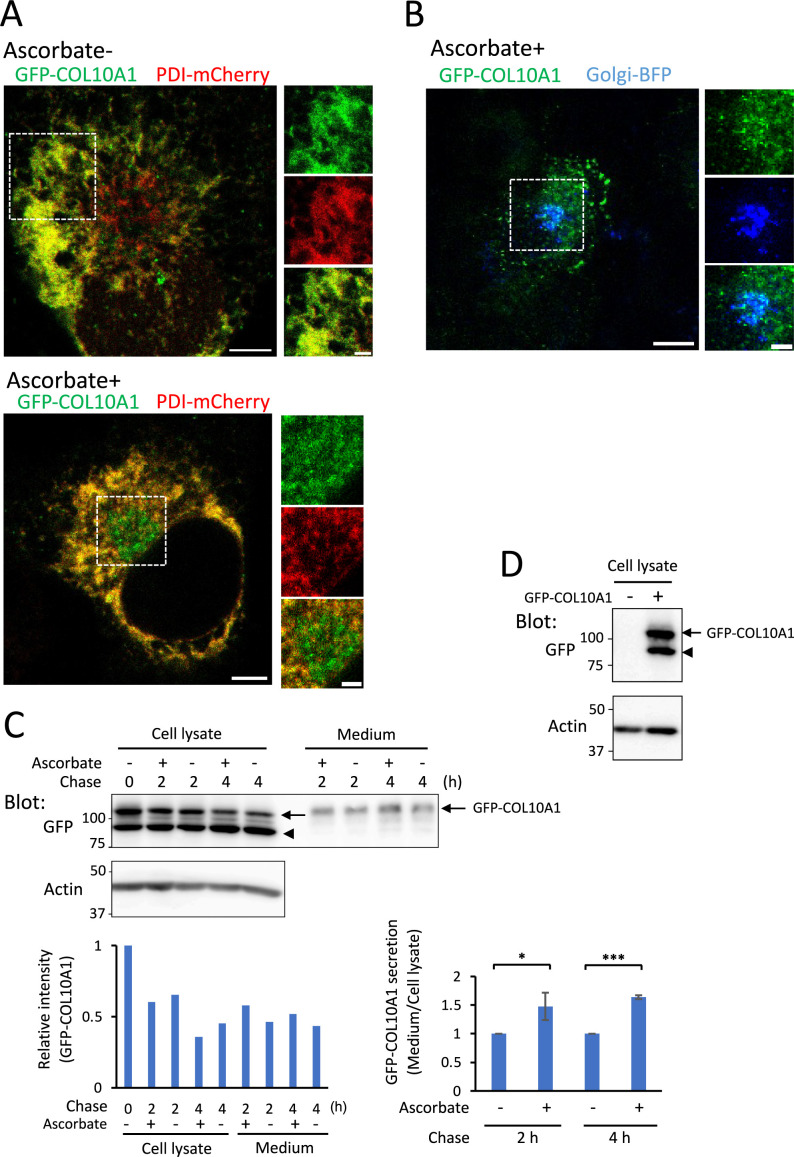
Expression and secretion of GFP-COL10A1 (A) Live-cell imaging of HT-1080 cells transiently expressing GFP-COL10A1 (green) and PDI-mCherry (red). Cells were cultured in the absence of ascorbate (Ascorbate –, upper panels) or after addition of ascorbate for 1 h (Ascorbate +, lower panels). Panels on the right show higher magnification images of the white dotted boxed regions. Scale bars, 5 μm (left panel) and 2 μm (right panels). (B) Live-cell imaging of HT-1080 cells transiently expressing GFP-COL10A1 (green) and Golgi-BFP (blue). Cells were cultured in the presence of ascorbate for 1 h. Panels on the right show higher magnification images of the white dotted boxed region. Scale bars, 5 μm (left panel) and 2 μm (right panels). (C) Western blot analysis of HT-1080 cells transiently expressing GFP-COL10A1. After incubation in the absence of ascorbate for 2 days, cells were cultured in SFM containing ascorbate and CHX for the indicated period. Proteins in the cell lysate and SFM were precipitated with TCA, dissolved in Laemmli’s buffer, separated by 12.5% SDS-PAGE, and analyzed by immunoblotting. The graphs show the relative intensity of GFP-COL10A1 (left panel) and the ratio of GFP-COL10A1 secreted into the medium (right panel, mean ± s.d. of three independent experiments). Actin was used as a loading control. Arrows indicate GFP-COL10A1. The arrowhead indicates the C-terminally truncated form of GFP-COL10A1. *P<0.05; ***P<0.001 (two-tailed Student’s *t*-test). (D) Immunoblotting of HT-1080 cells transiently expressing mock (indicated by –) or GFP-COL10A1 (indicated by +). The arrow and arrowhead indicate the same as in (C).

Procollagen X consists of a homotrimer of three α1 (X) chains (COL10A1). To analyze ER-to-Golgi transport of procollagen X in live cells, we constructed GFP-COL10A1, which expresses cfSGFP2 in the N-telopeptide region of COL10A1. We successfully monitored intracellular transport of GFP-COL3A1 (Hirata *et al.*, 2022) and GFP-COL4A1 (Matsui *et al.*, 2020) in live cells by introducing cfSGFP2 into the N-telopeptide regions of these procollagens, suggesting that the presence of the fluorescent tag in this domain does not inhibit triple helix formation. Using these constructs, we detected GFP-tagged collagens after secretion, even when the N-propeptides were cleaved. When GFP-COL10A1 and PDI-mCherry, which is an ER marker, were co-expressed in HT-1080 cells in the absence of ascorbate, GFP signals merged with mCherry signals during live-cell imaging (Fig. 1A, upper panel, quantified in Suppl. Fig. 1A), indicating GFP-COL10A1 was expressed in the ER. After ascorbate treatment for 1 h, GFP signals accumulated in a region adjacent to the nucleus, from which PDI-mCherry was excluded (Fig. 1A, lower panel, quantified in Suppl. Fig. 1A), suggesting GFP-COL10A1 was transported to the Golgi apparatus. As expected, GFP-COL10A1 co-localized with Golgi-BFP after ascorbate treatment for 1 h (Fig. 1B, quantified in Suppl. Fig. 1B).

We next analyzed secretion of GFP-COL10A1 by western blotting. Upon transient expression of GFP-COL10A1 in HT-1080 cells, the amount of GFP-COL10A1 in the cell lysate decreased during the chase period, with a reciprocal increase in the culture medium (Fig. 1C, indicated by arrows and quantified in the graph). Secretion of GFP-COL10A1 increased in the presence of ascorbate; however, a significant amount of GFP-COL10A1 was secreted into the medium even in the absence of ascorbate (Fig. 1C, Medium, Ascorbate – lanes). We also detected a GFP-positive signal smaller than the expected molecular weight of GFP-COL10A1, which was not secreted into the medium (Fig. 1C, indicated by arrowhead). This signal originated from GFP-COL10A1 (Fig. 1D), possibly due to truncation of the C-terminal region of type X collagen. The amount of this truncated form differed depending on the cell line transfected (see Fig. 7B for HeLa cells).

GFP-COL10A1 secreted into the medium was found as deposits in thin sponge-like or short rod-like structures in cultures of both HT-1080 and p52 cells (Suppl. Fig. 2A). The extracellular nature of these deposits was confirmed by co-staining of CRT with or without cell permeabilization (Suppl. Fig. 2B). Collectively, we confirmed that ectopically expressed GFP-COL10A1 was secreted and deposited in the extracellular space.

### ER-to-Golgi transport of GFP-COL10A1 by ERGIC membrane-containing vesicles

**Fig. 2:**
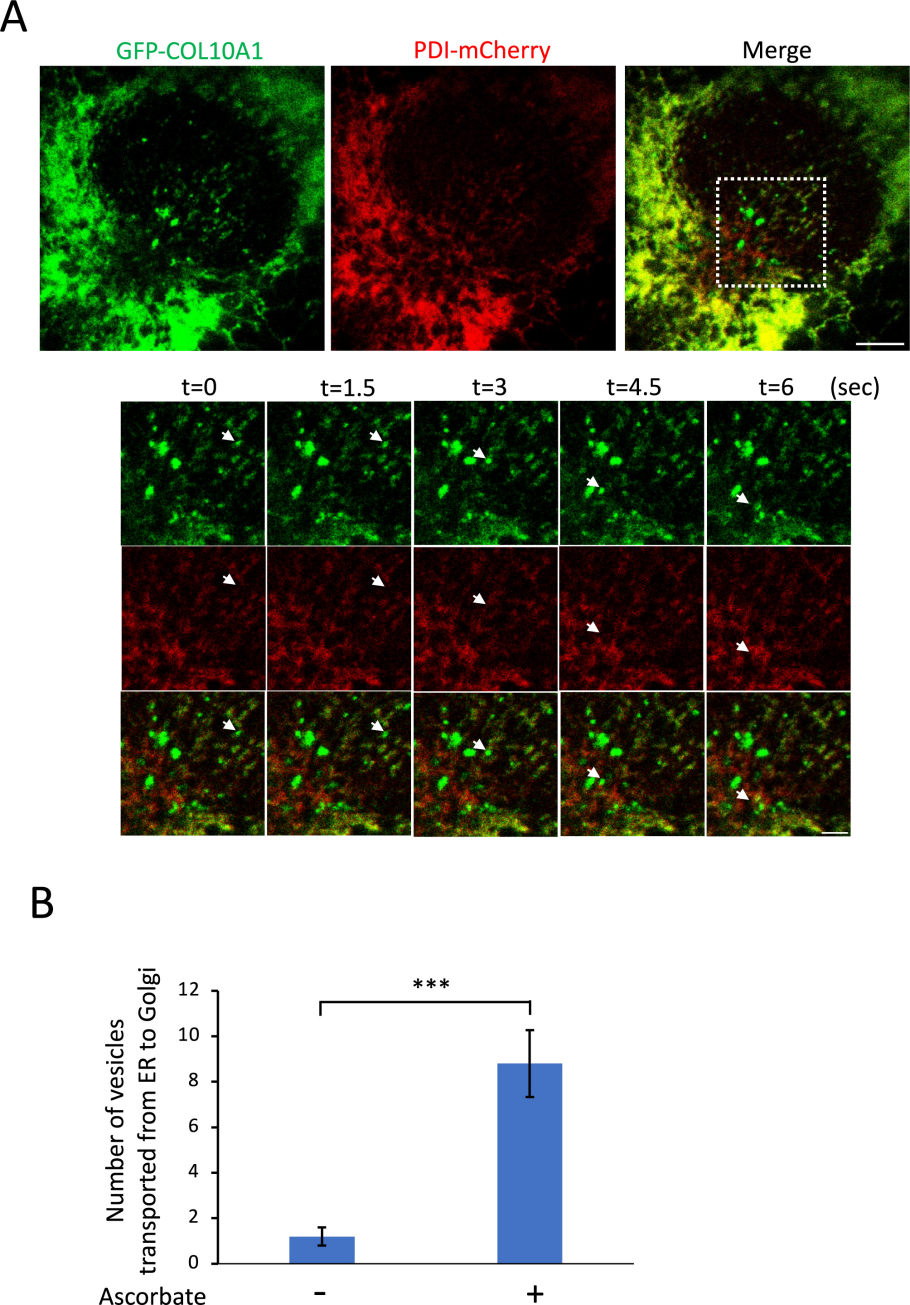
Visualization of ER-to-Golgi transport of GFP-COL10A1 (A) Live-cell imaging of HT-1080 cells transiently expressing GFP-COL10A1 (green) and PDI-mCherry (red) by confocal microscopy at ~40 min after addition of ascorbic acid. Time-lapse images were obtained every 1.5 sec for 3 min after photo-bleaching the Golgi area (lower panels, white dotted boxed region). Times denote the period after identification of a trafficking vesicle. Arrows indicate a GFP-COL10A1-containing vesicle trafficking from the ER to the Golgi. Scale bars, 5 μm (upper panels) and 2 μm (time-lapse). (B) Number of GFP-positive vesicles moving toward the Golgi complex during the 3 min of time-lapse imaging before (ascorbate –) and after (ascorbate +, 30–90 min after addition of ascorbate) addition of ascorbate. GFP-COL10A1-containing vesicles in five cells were counted in three independent experiments, and mean ± s.d. is shown. ***P<0.001 (two-tailed Student’s *t*-test).

To analyze transport of GFP-COL10A1 in HT-1080 cells, we co-﻿transfected GFP-COL10A1 and PDI-mCherry and performed live-﻿cell imaging. After addition of ascorbate, GFP-COL10A1-containing vesicles that lacked PDI-mCherry moved quickly to the Golgi apparatus (Fig. 2A and Movie 1, quantified in Fig. 2B). The movement of these vesicles toward the Golgi apparatus was confirmed by co-transfecting GFP-COL10A1 with Golgi-BFP (Suppl. Fig. 3 and Movie 2).

**Fig. 3:**
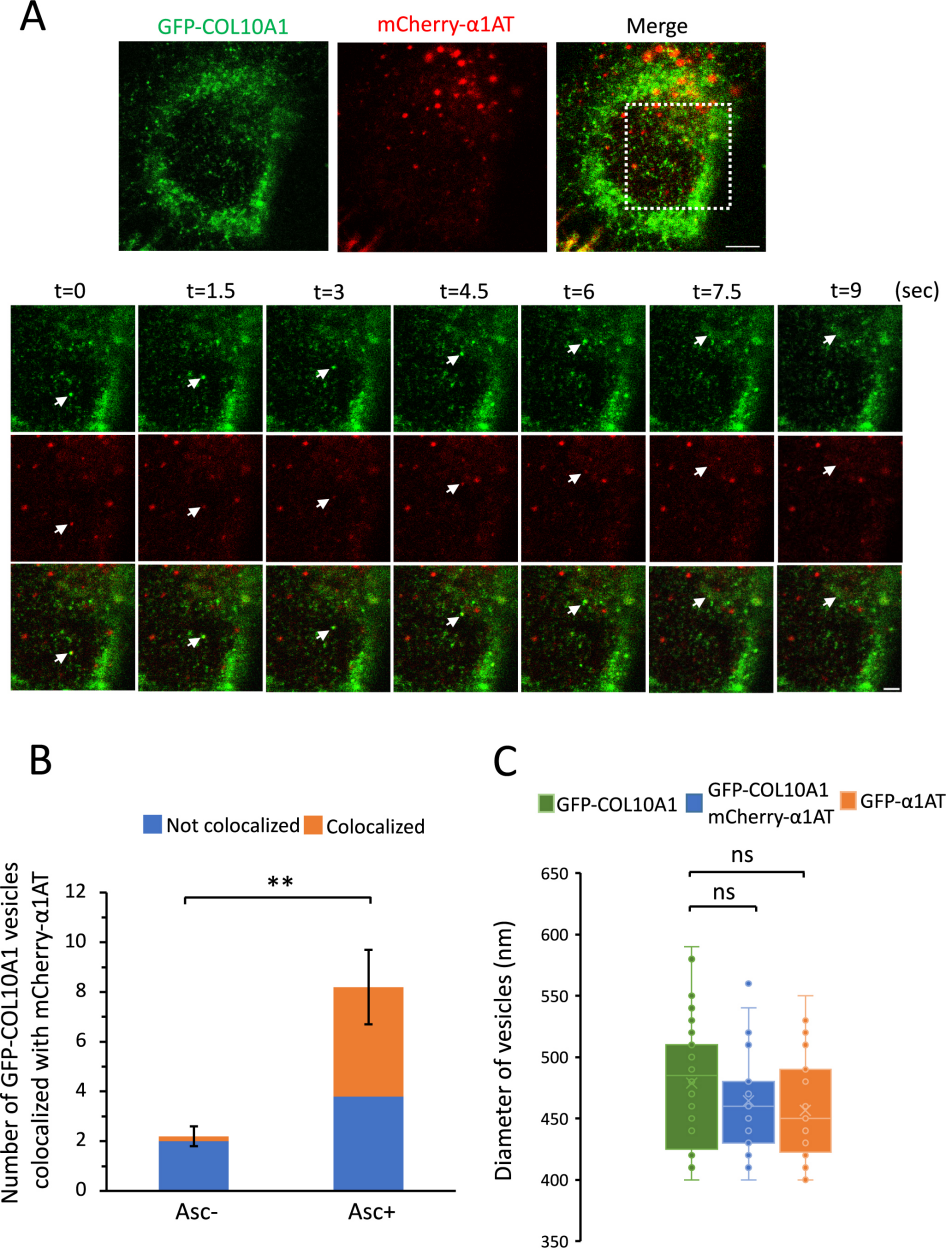
Co-transport of GFP-COL10A1 and mCherry-α1AT from the ER to the Golgi (A) Same as in Fig. 2A, except cells transiently expressed GFP-COL10A1 (green) and mCherry-α1AT (red). (B) Same as in Fig. 2B, except the number of GFP-positive vesicles that co-localized or did not co-localize with mCherry-α1AT was counted. Mean ± s.d. of GFP-COL10A1-containing vesicles (n = 11 and 44 under ascorbate (–) and ascorbate (+) conditions, respectively) in five cells from three independent experiments. **P<0.01 (two-tailed Student’s *t*-test). (C) Diameters of vesicles containing GFP-COL10A1, both GFP-COL10A1 and mCherry-α1AT, and GFP-α1AT transported to the Golgi. Cells transiently expressing GFP-COL10A1, both GFP-COL10A1 and mCherry-α1AT, and GFP-α1AT were treated with ascorbate. Vesicles (n = 28, 26, and 28 for GFP-COL10A1, both GFP-COL10A1 and mCherry-α1AT, and GFP-α1AT, respectively) from three independent experiments were measured. ns, not significant (Dunnett’s test).

We previously showed that GFP-COL3A1 is co-transported from the ER to the Golgi apparatus with the conventional cargo α1AT (Hirata *et al.*, 2022). To determine whether GFP-COL10A1 is also co-transported with such cargoes, we co-transfected GFP-COL10A1 and mCherry-α1AT into HT-1080 cells. More than half of GFP-COL10A1-containing vesicles were positive for mCherry (Fig. 3A and Movie 3, quantified in Fig. 3B), indicating that GFP-COL10A1 was mostly co-transported with the conventional cargo α1AT. The mean diameters of vesicles carrying only GFP-COL10A1, both GFP-COL10A1 and mCherry-α1AT, and GFP-α1AT alone were 479, 464, and 456 nm, respectively, and did not significantly differ (Fig. 3C).

**Fig. 4:**
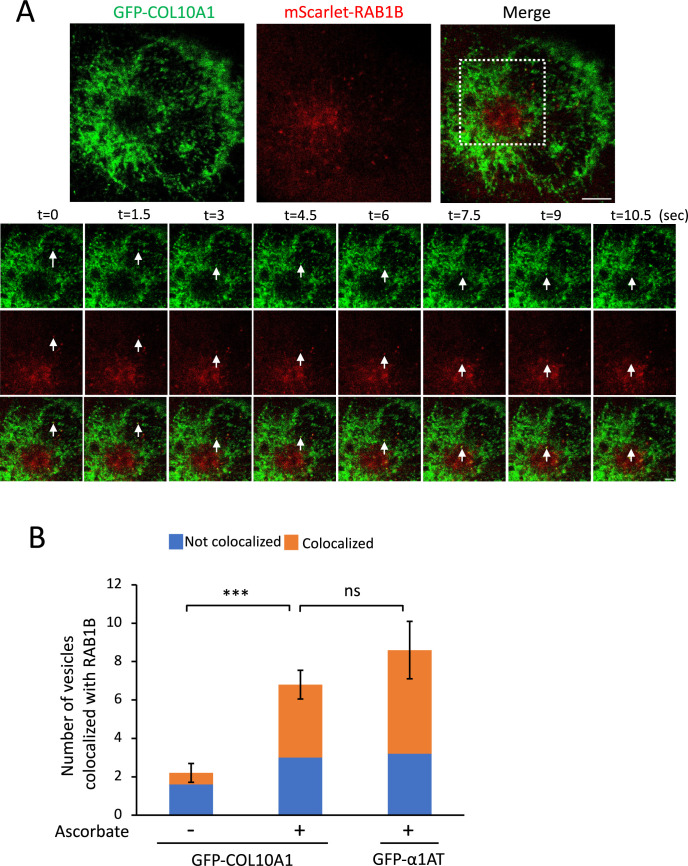
GFP-COL10A1-carrying vesicles contain ERGIC membranes (A) Same as in Fig. 2A, except cells transiently expressed GFP-COL10A1 (green) and mScarlet-RΑΒ1Β (red). (B) Same as in Fig. 2B, except the number of GFP-positive vesicles that co-localized or did not co-localize with mScarlet-RΑΒ1Β was counted. The number of GFP-α1AT-containing vesicles was counted in cells transiently expressing GFP-α1AT in the presence of ascorbate. Mean ± s.d. of vesicle numbers (n = 11, 34, and 43 for GFP-COL10A1 without ascorbate, GFP-COL10A1 with ascorbate, and GFP-α1AT with ascorbate, respectively) in five cells (per condition) from three independent experiments. Error bars denote the s.d. of three independent experiments. ***P<0.001; ns, not significant (two-tailed Student’s *t*-test).

To confirm that GFP-COL10A1-carrying vesicles contain ERGIC membranes, we co-expressed GFP-COL10A1 and mScarlet-RAB1B/mCherry-ERGIC53, which are ERGIC marker proteins (Hauri *et al.*, 2000; Sannerud *et al.*, 2006). After addition of ascorbate, GFP-COL10A1-carrying vesicles started to move toward the Golgi apparatus and more than half co-localized with mScarlet-RAB1B (Fig. 4A and Movie 4, quantified in Fig. 4B). A similar degree of colocalization with mScarlet-RAB1B was observed for GFP-α1AT-bearing vesicles (Fig. 4B). GFP-COL10A1 was also incorporated in tubular structures (17.7% in cells co-transfected with mScarlet-RAB1B; Suppl. Fig. 4 and Movie 5), as observed during ER-to-Golgi transport of GFP-COL3A1 (Hirata *et al.*, 2022). mCherry-ERGIC53 was also incorporated into GFP-COL10A1-containing vesicles (Suppl. Fig. 5 and Movie 6). These observations indicate that GFP-COL10A1 is transported from the ER to the Golgi apparatus via tubulo-vesicular carriers that contain ERGIC membranes (Presley *et al.*, 1997; Watson and Stephens, 2005; Brandizzi and Barlowe, 2013) with a conventional cargo.

### Formation of large droplets of procollagen X prior to ER exit via ERES

**Fig. 5:**
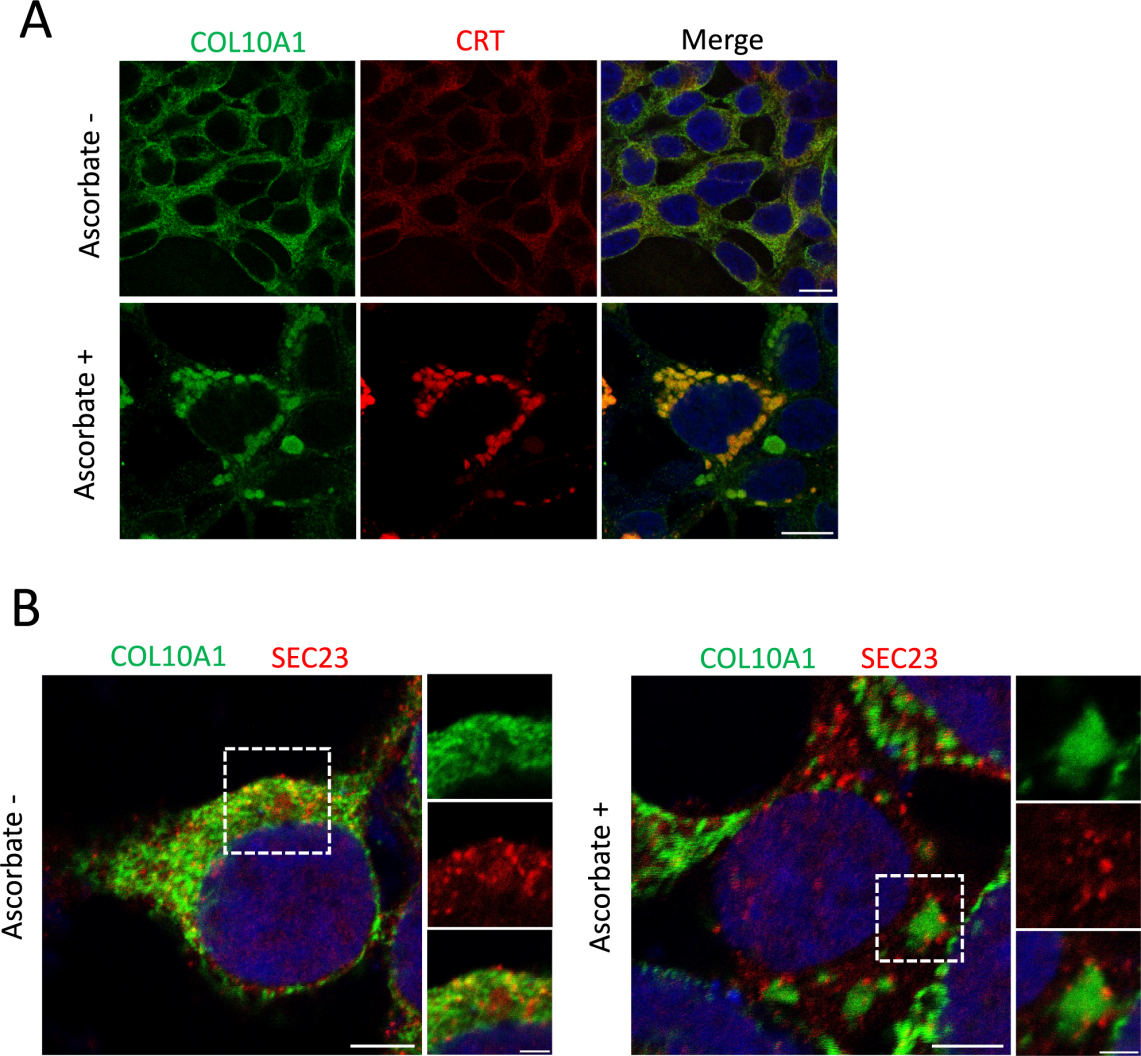
Localization of SEC23 around the GFP-COL10A1 condensation in the ER (A) Immunostaining of p52 cells. Cells were cultured in the absence of ascorbate for 2 days. After incubation with or without ascorbate for 4 h, cells were fixed and stained with anti-type X collagen (green) and anti-CRT (red) antibodies. Scale bars, 5 μm. (B) Same as in (A), except anti-type X collagen (green) and anti-SEC23 (red) antibodies were used. Panels on the right show higher magnification images of the white dotted boxed regions. Scale bars, 10 μm (left panel) and 2 μm (right panels).

p52 cells were fixed and immunostained to analyze whether GFP-COL10A1 was exported from ERES. Procollagen X was distributed in a fine reticular network in the ER, which co-localized with CRT in the absence of ascorbate (Fig. 5A, Ascorbate –). After addition of ascorbate, procollagen X formed droplet-like condensations in the ER, which were also labeled with an anti-CRT antibody (Fig. 5A, Ascorbate +). These condensations were similar to those observed for procollagen III in RD cells (Hirata *et al.*, 2022), suggesting that formation of liquid-liquid phase separation-like droplets is a common feature of procollagens prior to ER exit.

To determine whether procollagen X condensed in the droplets was released from ERES, we performed co-staining for procollagen X and SEC23, a marker of ERES. SEC23 was scattered throughout the ER in the absence of ascorbate, but was distributed around the procollagen X condensations after addition of ascorbate (Fig. 5B). These results suggest that procollagen X forms large droplet-like structures before it exits the ER via ERES, similar to procollagen III.

### Requirement of SAR1, SLY1/SCFD1, TANGO1L, and BET3/TRAPPC3 for export of procollagen X from the ER

**Fig. 6:**
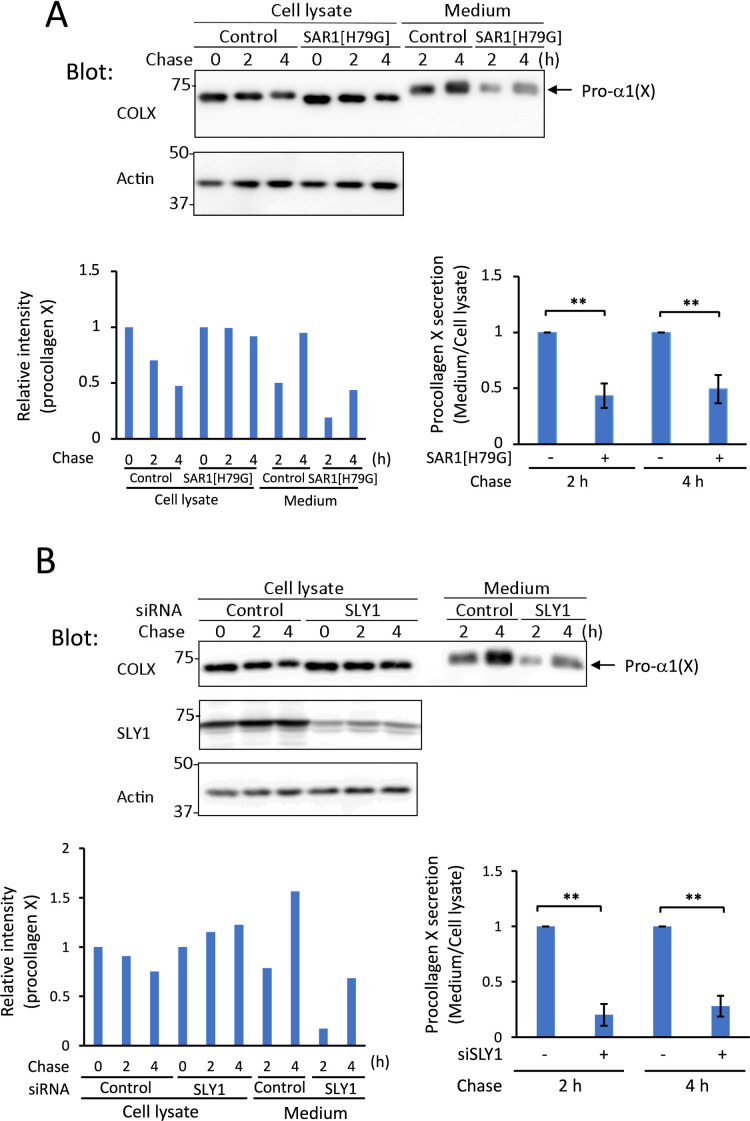
SAR1 and SLY1/SCFD1 are required for secretion of procollagen X (A) Western blot analysis of p52 cells. Twenty-four hours after transfection of the Sar1[H79G] mutant, cells were cultured in SFM containing CHX and ascorbate, and were chased for the indicated periods. After TCA precipitation of the cell lysate and culture medium, procollagen X was analyzed by western blotting. Actin was used as a loading control. The relative intensity of procollagen X was quantified (left graph). The ratio of procollagen X secreted into the medium was normalized to that in mock-transfected (Control) cells (right graph). Mean ± s.d. of three independent experiments. **P<0.01 (two-tailed Student’s *t*-test). (B) Same as in (A), except p52 cells were harvested 48 h after treatment with negative control siRNA (Control) or siRNA targeting SLY1/SCFD1. Procollagen X in the cell lysate and culture medium was analyzed by western blotting. Expression of SLY1/SCFD1 was also analyzed by immunoblotting. **P<0.01 (two-tailed Student’s *t*-test).

Our previous study revealed the distinct molecular machinery required for ER export of procollagens III and IV. SAR1, TANGO1, and CUL3 are required for procollagen III transport in RD cells (Hirata *et al.*, 2022), whereas SAR1 and SLY1/SCFD1 are needed for ER exit of procollagen IV in HT-1080 cells (Matsui *et al.*, 2020). To identify the proteins necessary for export of procollagen X from the ER, we transfected p52 cells with the SAR1[H79G] dominant negative mutant or siRNAs for other candidate proteins. SAR1 is a GTPase required for budding of COPII vesicles from ERES (reviewed by Miller and Barlowe, 2010; Van der Verren and Zanetti, 2023). Transient expression of SAR1[H79G] inhibited secretion of procollagen X (Fig. 6A). Silencing of SLY1/SCFD1, which is required for ER-to-Golgi transport of procollagens VII (Nogueira *et al.*, 2014), II (Hou *et al.*, 2017), and IV (Matsui *et al.*, 2020), effectively diminished secretion of procollagen X (Fig. 6B). A component of the TRAPPII complex, sedlin/TRAPPC2, is needed for transport of procollagens I and II (Venditti *et al.*, 2012). Hence, we used siRNA targeting BET3/TRAPPC3, another subunit of the TRAPP complex (Barrowman *et al.*, 2010; Kim *et al.*, 2016), and observed that it mildly inhibited procollagen X secretion (Suppl. Fig. 6A and E). ZW10 is a component of the NRZ tethering complex required for ER-to-Golgi transport of procollagen VII (Raote *et al.*, 2018) and STX18 is one of the ER t-SNAREs required for procollagen VII secretion (Nogueira *et al.*, 2014). In addition, CUL3 is a scaffold protein of ubiquitin ligase necessary for secretion of procollagens I (Jin *et al.*, 2012) and III (Hirata *et al.*, 2022). However, siRNAs targeting ZW10, STX18, and CUL3 did not affect procollagen X secretion (Suppl. Fig. 6B–E).

**Fig. 7:**
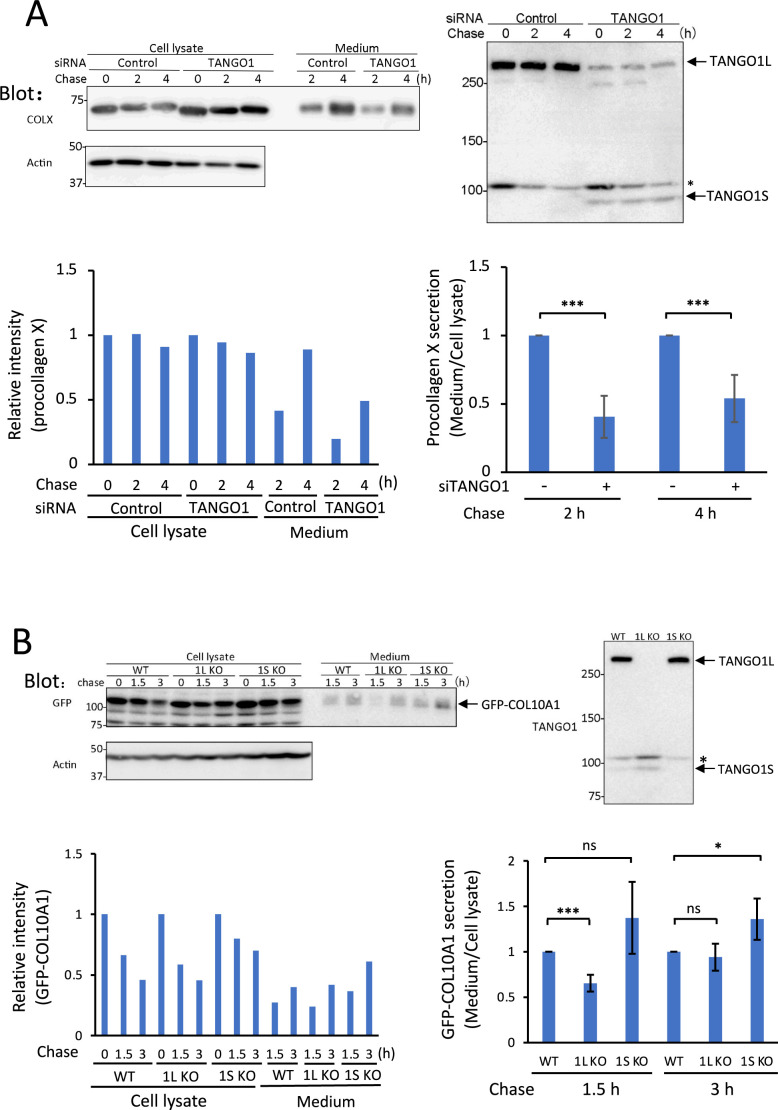
Requirement of TANGO1L and TANGO1S for secretion of procollagen X (A) Same as in Fig. 6A, except p52 cells were harvested 48 h after treatment with siRNA targeting TANGO1. The asterisk indicates a signal non-specifically detected by the anti-TANGO1 antibody. ***P<0.001 (two-tailed Student’s *t*-test). (B) Same as in (A), except HeLa cells (WT, 1L KO, and 1S KO cells) were analyzed 48 h after transfection of GFP-COL10A1. GFP-COL10A1 in the cell lysate and culture medium was assessed by western blotting. *P<0.05; ***P<0.001; ns, not significant (two-tailed Student’s *t*-test).

TANGO1 is an important molecule required for secretion of procollagens (reviewed by Raote and Malhotra, 2021; Aridor, 2022). The two splicing variants, TANGO1L and TANGO1S, which lacks the N-terminal half of TANGO1L, have been well-characterized (Wilson *et al.*, 2011). Silencing of TANGO1L reciprocally upregulates TANGO1S (Maeda *et al.*, 2016). The siRNA we used to target TANGO1 (Santos *et al.*, 2015), which specifically recognizes TANGO1L, inhibited procollagen X secretion in p52 cells (Fig. 7A). Upon treatment with this siRNA, TANGO1L was efficiently downregulated whereas TANGO1S was greatly upregulated (Fig. 7A). To discriminate the effect of TANGO1L from that of TANGO1S on procollagen X secretion, we established HeLa cells that specifically lack TANGO1L (TANGO1L-KO) or TANGO1S (TANGO1S-KO). Secretion of GFP-COL10A1 was inhibited during the early chase period in TANGO1L-KO cells, but was enhanced in TANGO1S-KO cells (Fig. 7B). TANGO1S was upregulated in TANGO1L-KO cells, while the expression level of TANGO1L was similar in TANGO1S-KO and WT cells (Fig. 7B). To confirm the specificity of TANGO1L and TANGO1S for procollagen X secretion in each KO cell line, we transduced TANGO1L-HA or TANGO1S-HA into KO cells using lentivirus. Expression of TANGO1S-HA suppressed secretion of GFP-COL10A1 in TANGO1S-KO Hela cells (Suppl. Fig. 7), indicating that TANGO1S inhibits procollagen X secretion. Although TANGO1L-HA was transduced into TANGO1L-KO cells, we could not detect its expression, probably due to the low transduction efficiency of TANGO1L, which consists of 1,907 amino acids (data not shown). Secretion of α1AT, which was used as a model conventional cargo, was unchanged in TANGO1L- and TANGO1S-KO cells (Suppl. Fig. 8). We conclude that SAR1, SLY1/SCFD1, TANGO1L and BET3/TRAPPC3 are required for secretion of procollagen X, and that different proteins are needed for secretion of different procollagens.

## Discussion

To comprehensively understand ER-to-Golgi transport of various types of procollagens, we analyzed the trafficking of procollagen X in the present study. We found that GFP-COL10A1 was transported by conventional vesicular and tubular carriers containing ERGIC membranes. The diameter of the transport vesicles was 400–550 nm (Fig. 3C), which is similar to those of transport vesicles of procollagens IV (300–500 nm) (Matsui *et al.*, 2020) and III (350–400 nm) (Hirata *et al.*, 2022). Conventional cargoes exit the ER in COPII vesicles with a diameter of 60–80 nm and fuse to the ERGIC shortly thereafter (Zanetti *et al.*, 2011; Peotter *et al.*, 2019). Considering that the procollagen X trimer is 100–130 nm long (Schmid *et al.*, 1984; Kwan *et al.*, 1991; Frischholz *et al.*, 1998), procollagen X is likely incorporated into COPII vesicles that transport conventional cargoes such as α1AT from ERES (Suppl. Fig. 9). Thus, we propose that the ER-to-Golgi transport pathways of procollagens III, IV, and X differ. Specifically, procollagen III exits the ER in enlarged COPII vesicles, which also accommodate ordinary cargoes and fuse with the ERGIC; procollagen IV exits the ER in enlarged COPII vesicles, but is delivered by carriers that lack ERGIC membranes; and procollagen X possibly uses the same transport pathway as conventional cargoes. To estimate the size of transport vesicles more accurately, application of more sophisticated methods is required in future.

We also revealed that different sets of proteins are required for ER-to-Golgi trafficking of procollagens III, IV, and X. In addition to SAR1, TANGO1 and CUL3 are necessary for trafficking of procollagen III in RD cells, SLY1/SCFD1 is required for trafficking of procollagen IV in HT-1080 cells, and TANGO1L, SLY1/SCFD1, and BET3/TRAPPC3 are needed for trafficking of procollagen X in p52 cells. CUL3, which is involved in ubiquitination and generates large COPII vesicles for transport of procollagen I (Jin *et al.*, 2012), is required for export of procollagen III, but not of procollagens IV and X. SLY1/SCFD1, which regulates membrane fusion of SNAREs (Carr and Rizo, 2010), is required for ER-to-Golgi transport of many secretory proteins (Bassaganyas *et al.*, 2019). We found that SLY1/SCFD1 is necessary for export of procollagens IV and X, and is also reportedly required for export of procollagens II (Hou *et al.*, 2017) and VII (Nogueira *et al.*, 2014), but not of procollagens I (Nogueira *et al.*, 2014) and III (Hirata *et al.*, 2022). BET3/TRAPPC3 is a component of the TRAPP complex, which acts as a guanine nucleotide exchange factor and tethers COPII vesicles to the ERGIC/vesicular-tubular cluster and Golgi (Barrowman *et al.*, 2010; Kim *et al.*, 2016). BET3/TRAPPC3 is required for transport of procollagen X as shown here, but not of procollagens III and IV. Sedlin/TRAPPC2, another component of the TRAPP complex, is necessary for ER-to-Golgi trafficking of procollagens I and II (Venditti *et al.*, 2012). Collectively, proteins required for ER-to-Golgi transport of procollagens seem to differ depending on the type of procollagen, most likely to accommodate their variable sizes. A limitation of this study is the use of several cell lines to analyze the export mechanism of type X procollagen, which might partly affect cell-type specificity of the machinery. Also, we do not rule out the possibility that knocked-down by siRNAs might have affected the secretory pathway other than ER-to-Golgi transport, since SLY1/SCFD1 may affect intra-Golgi transport (Laufman *et al.*, 2009) and retrograde transport from the Golgi (Li *et al.*, 2005) as well as autophagy (Huang *et al.*, 2021), and BET3 may also have contributed to intra-Golgi traffic (Kim *et al.*, 2005; Kummel *et al.*, 2006) and autophagy (Zou *et al.*, 2015).

We also analyzed the requirement of TANGO1L and TANGO1S for trafficking of procollagen X in p52 and HeLa cells. Knockdown of TANGO1L upregulates TANGO1S, and both isoforms accelerate secretion of procollagen VII (Maeda *et al.*, 2016). Our analysis was consistent with these results because silencing of TANGO1L upregulated TANGO1S (Fig. 7A and B). However, in HeLa cells, secretion of GFP-COL10A1 was inhibited by depletion of TANGO1L, but enhanced by depletion of TANGO1S (Fig. 7B). TANGO1S is transcribed from exon 1B in the sixth intron (Wilson *et al.*, 2011) and thus lacks the N-terminal half of TANGO1L, which contains the collagen-interacting SH3 domain (Saito *et al.*, 2009; Ishikawa *et al.*, 2016). The reason why TANGO1S has different effects on secretion of procollagens VII and X is unclear at present. Of note, McCaughey and coworkers reported that simultaneous depletion of TANGO1L and TANGO1S severely affects protein secretion by disrupting cellular integrity (McCaughey *et al.*, 2021).

Interestingly, condensation of procollagen X in the ER was observed in p52 cells prior to ER exit (Fig. 5), but not in HT-1080 cells, which produce smaller amount of GFP-procollagen X. A similar phenomenon was observed for procollagens III (Hirata *et al.*, 2022) and II (Pacifici and Iozzo, 1988) after addition of ascorbate. Thus, procollagen condensation in the ER may be a common phenomenon when the expression levels of procollagens are high.

It should be noted that we detected a considerable amount of secreted GFP-COL10A1 even in the absence of ascorbate by western blotting (Fig. 1C). Although live-cell imaging revealed that GFP-COL10A1-containing vesicles rapidly moved from the ER to the Golgi apparatus only after addition of ascorbate (Fig. 2A), with a greater than 8-fold increase in the number of vesicles (Fig. 2B), the reason for this high constitutive secretion requires further analysis.

## Funding

This work was supported by a grant-in-aid for Scientific Research (KAKENHI) from the Ministry of Education, Culture, Sports, Science, and Technology of Japan (18K06214 to N.H. and 17K07311 to I.W.).

## Abbreviations

α1AT: α_1_-antitrypsin
CHX: cycloheximide
COPII: coat protein complex II
CRT: calreticulin
ER: endoplasmic reticulum
ERES: ER exit site
ERGIC: ER-Golgi intermediate compartment
KO: knockout
s.d.: standard deviation
SFM: serum-free medium
siRNA: small interfering RNA
WT: wild-type

## Supplementary Material

Movie 1. Live-cell imaging of HT-1080 cells transiently expressing GFP-COL10A1 (green) and PDI-mCherry (red). After addition of ascorbic acid, the Golgi region was photo-bleached. Time-lapse images were acquired every 1.5 sec using a confocal microscope (Leica SP8). The time point at which the vesicle was identified was set to 0.0 sec. Arrows indicate a GFP-COL10A1-containing vesicle trafficking from the ER to the Golgi. Individual time-lapse images are shown in Figure 2A. Scale bar, 2 μm.

Movie 2. Same as in Movie 1, except live-cell imaging of HT-1080 cells transiently expressing GFP-COL10A1 (green) and Golgi-BFP (blue) was performed. Arrows indicate a GFP-COL10A1-carrying vesicle moving from the ER to the Golgi. Individual time-lapse images are shown in Suppl. Fig. 3. Scale bars, 2 μm.

Movie 3. Same as in Movie 1, except live-cell imaging of HT-1080 cells transiently expressing GFP-COL10A1 (green) and mCherry-α1AT (red) was performed. Arrows indicate a vesicle carrying both GFP-COL10A1 and mCherry-α1AT moving from the ER to the Golgi. Individual time-lapse images are shown in Figure 3A. Scale bars, 2 μm.

Movie 4. Same as in Movie 1, except live-cell imaging of HT-1080 cells transiently expressing GFP-COL10A1 (green) and mScarlet-RAB1B (red) was performed. Arrows indicate a vesicle positive for both COL10A1 and mScarlet-RAB1B trafficking from the ER to the Golgi. Individual images are shown in Figure 4A. Scale bars, 2 μm.

Movie 5. Same as in Movie 4, except arrows indicate a tubular structure carrying both COL10A1 and mScarlet-RAB1B trafficking from the ER to the Golgi. Individual images are shown in Suppl. Fig. 4. Scale bars, 2 μm.

Movie 6. Same as in Movie 1, except live-cell imaging of HT-1080 cells transiently expressing GFP-COL10A1 (green) and mCherry-ERGIC53 (red) was performed. Arrows indicate a vesicle positive for both COL10A1 and mCherry-ERGIC53 moving from the ER to the Golgi. Individual images are shown in Suppl. Fig. 5. Scale bars, 2 μm.
