# Supplementary material for: Visualization of ER-to-Golgi trafficking of procollagen X
Source: Cell Struct Funct. 2024 Sep 6;49(2):67–81. doi: 10.1247/csf.24024 (PMC11930776; doi:10.1247/csf.24024)
Supplement: Supplementary file 7 — Supplementary Materials [file csf_49_24024_7.zip › 49_24024_1.pdf]

Suppl. Fig. 1

A

Ascorbate-

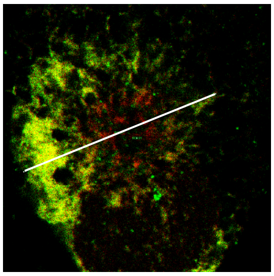

GFP-COL10A1
PDI-mCherry

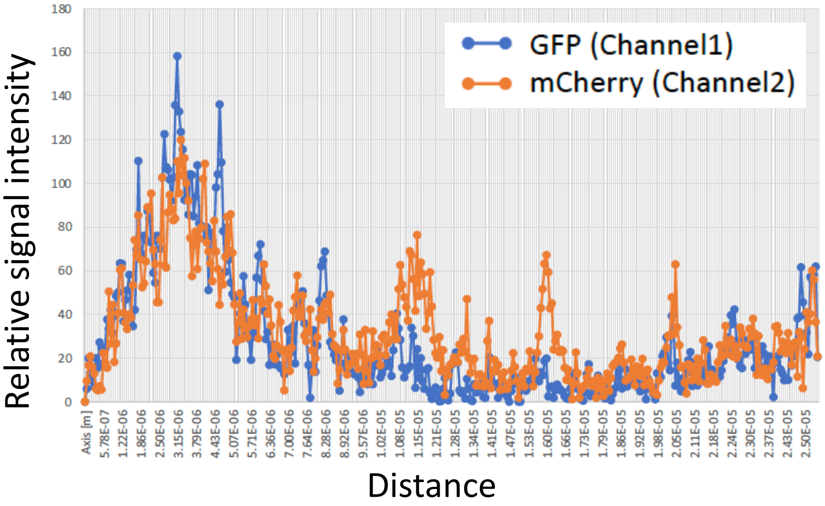

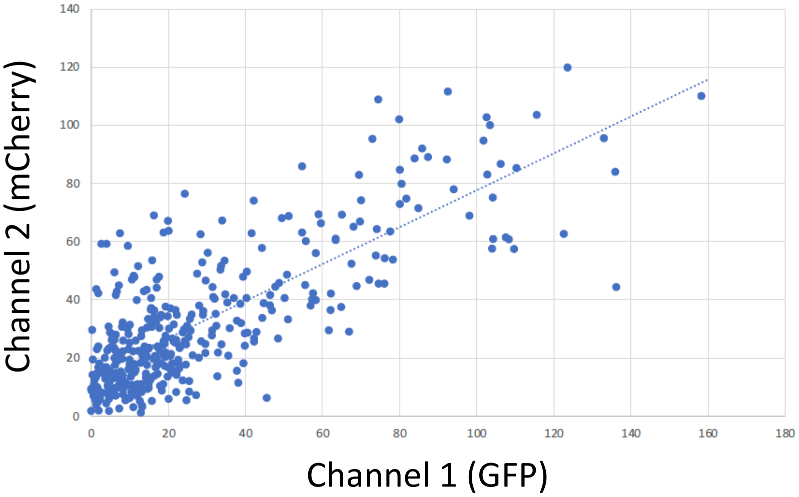

Pearson correlation coefficient=0.78

Ascorbate+

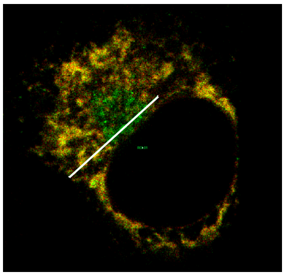

GFP-COL10A1
PDI-mCherry

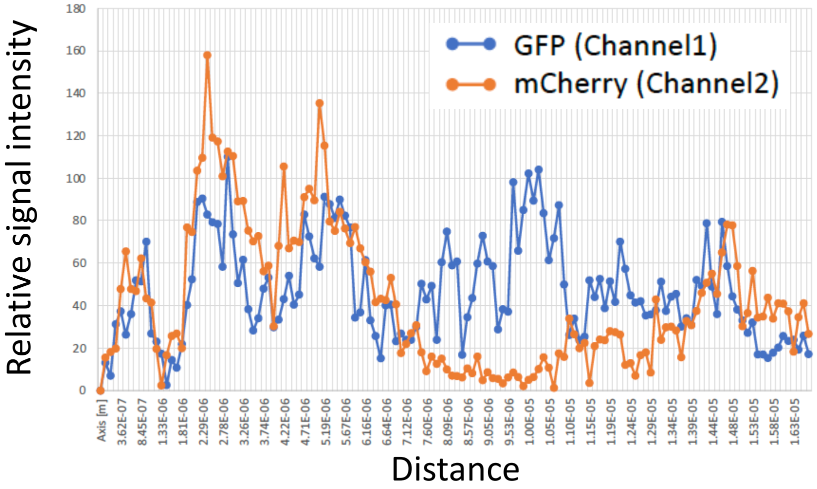

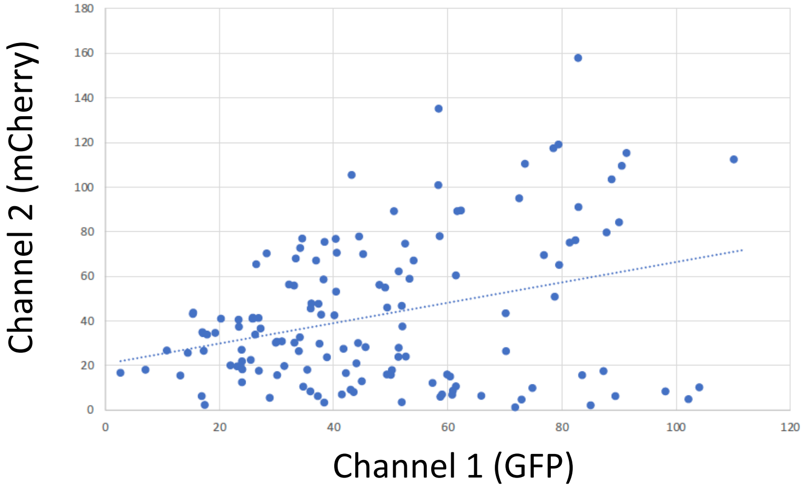

Pearson correlation coefficient=0.32

Suppl. Fig. 1

B

Ascorbate+

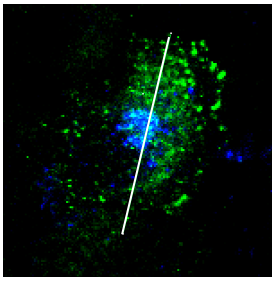

GFP-COL10A1
Golgi-BFP

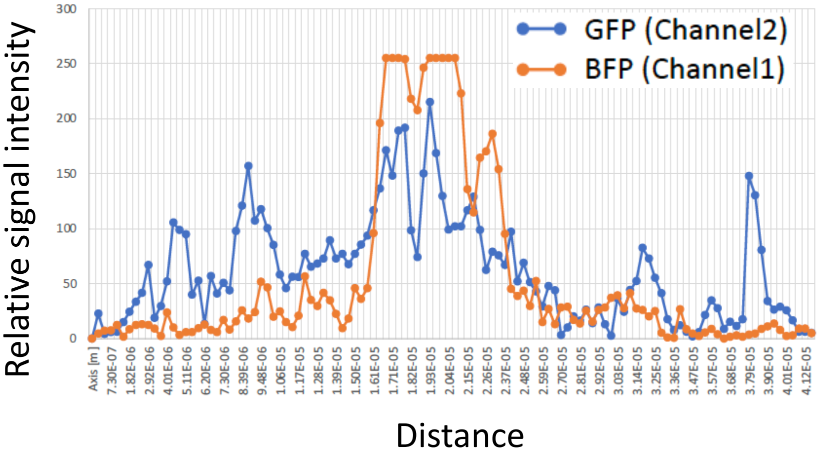

Pearson correlation coefficient=0.69

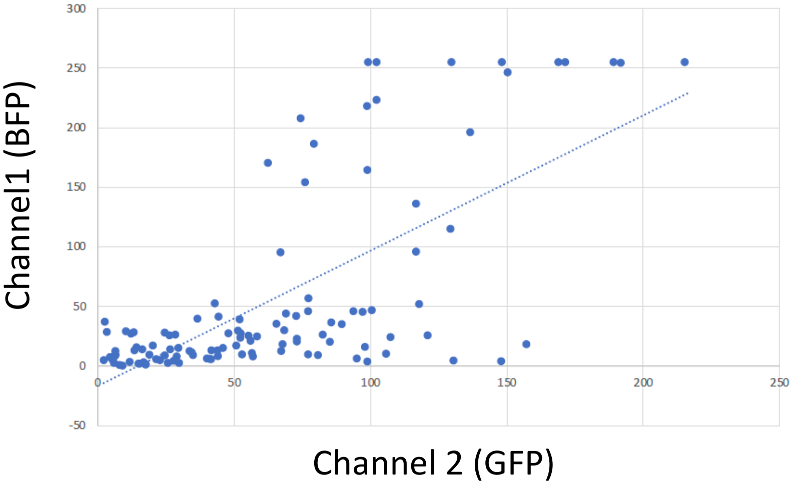

Ascorbate+, Golgi

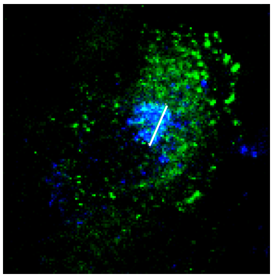

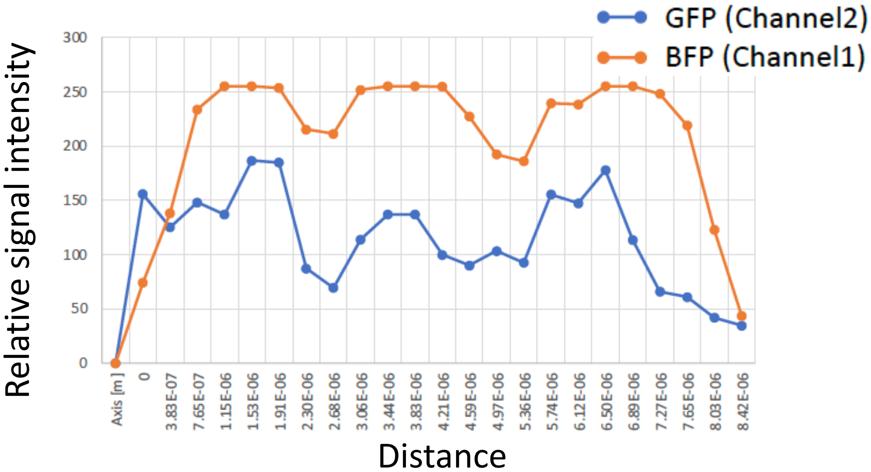

Ascorbate+, ER

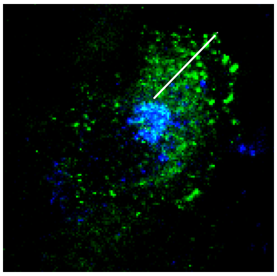

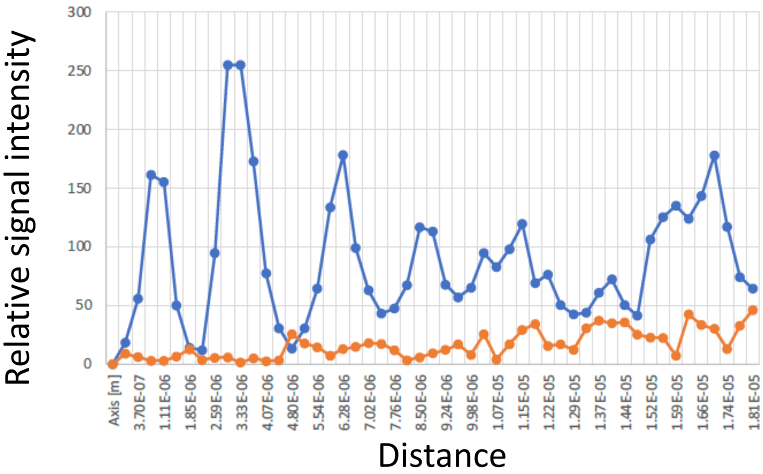

Suppl. Fig. 2

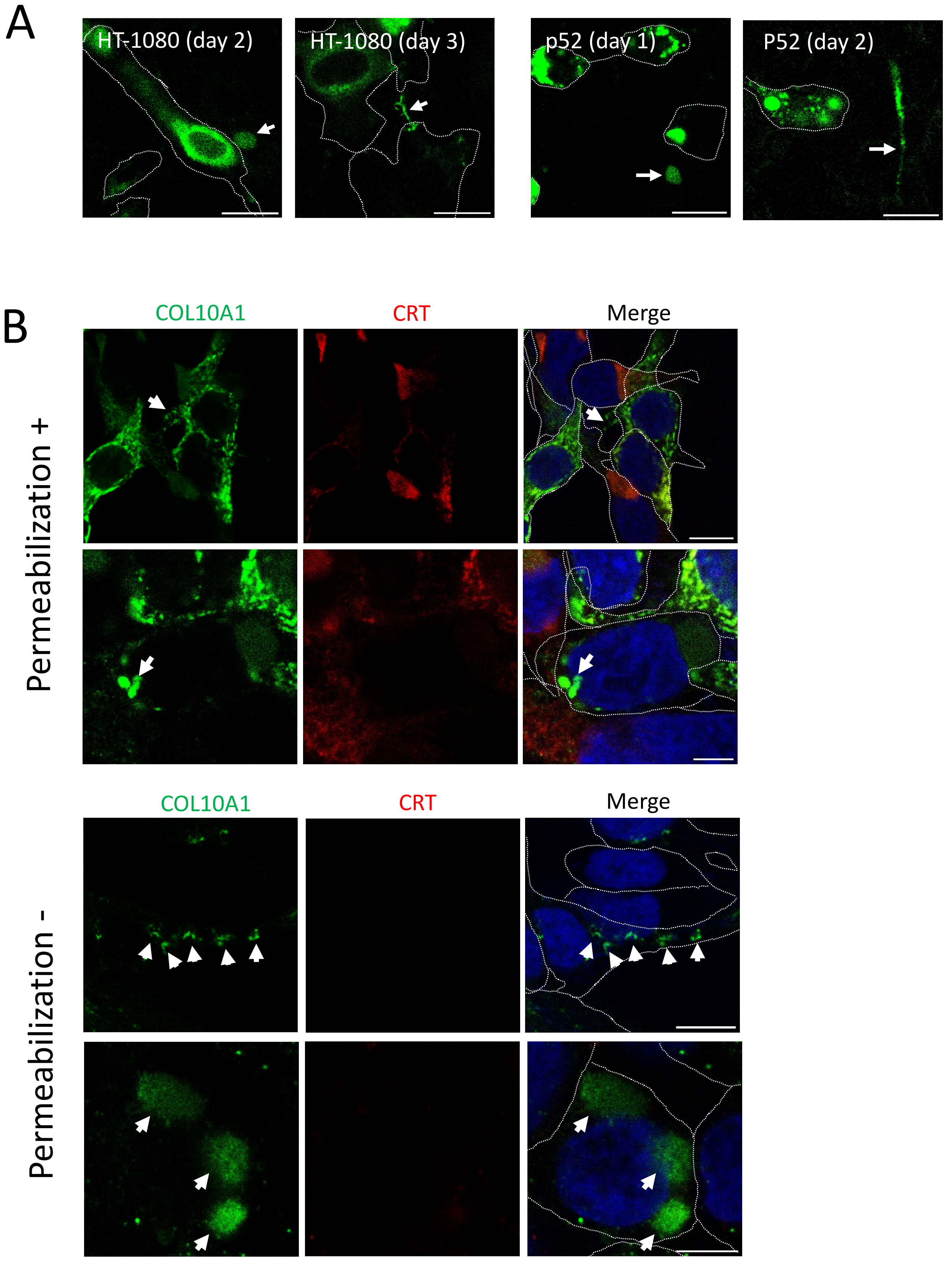
A
HT-1080 (day 2)
HT-1080 (day 3)
p52 (day 1)
P52 (day 2)
B
COL10A1
CRT
Merge
Permeabilization +
COL10A1
CRT
Merge
Permeabilization -

Suppl. Fig. 3

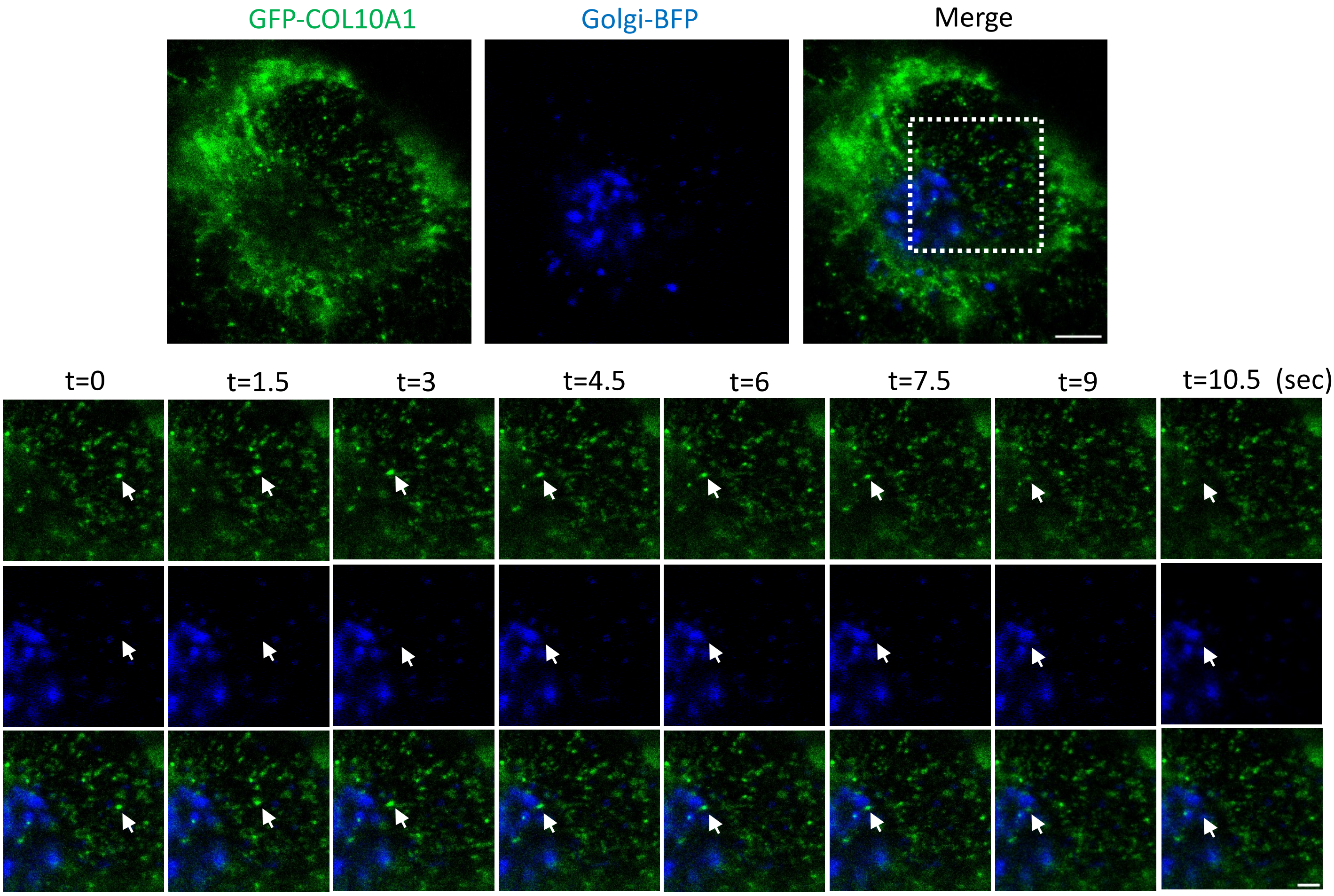

Suppl. Fig. 4

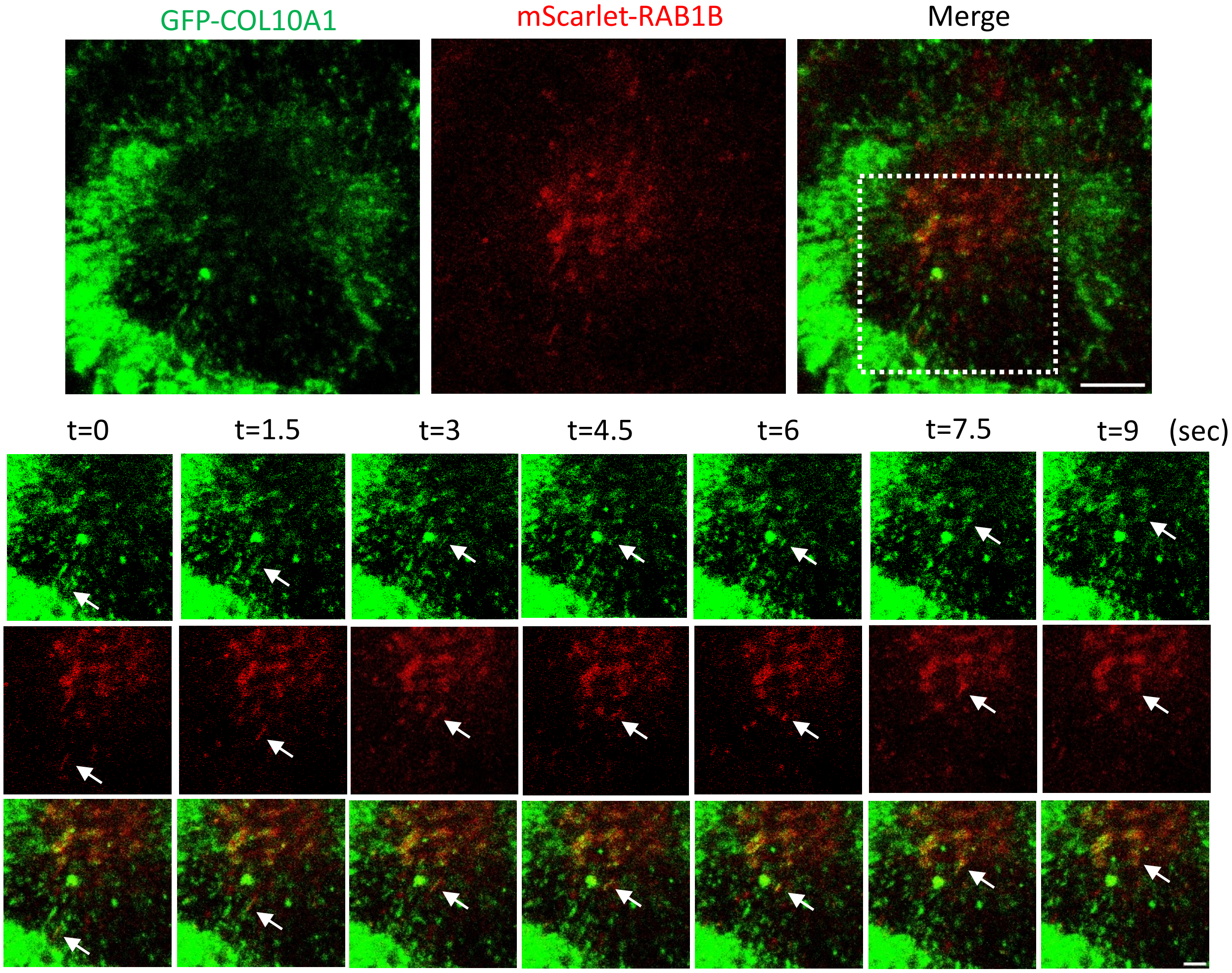

Suppl. Fig. 5

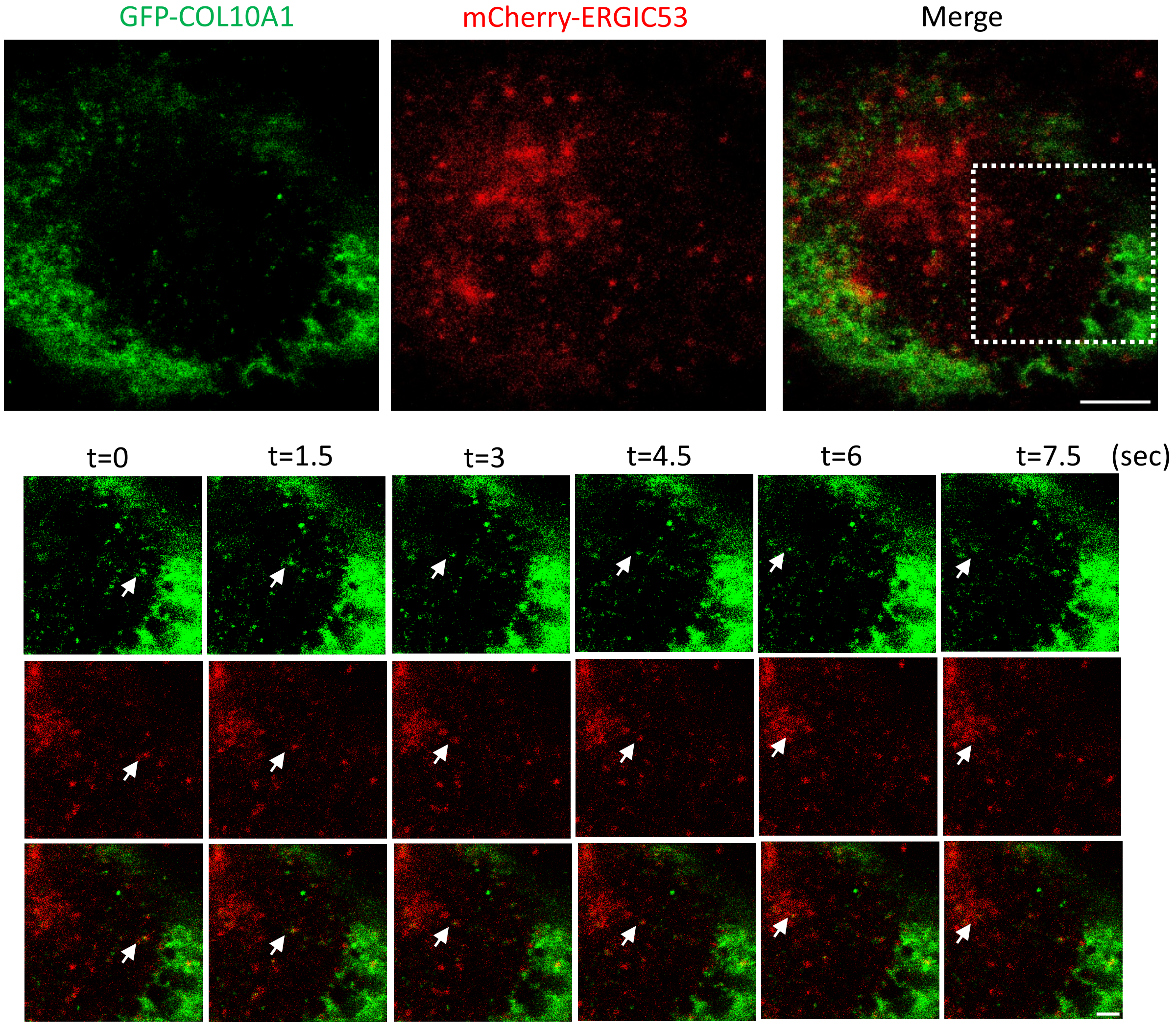

Suppl. Fig. 6

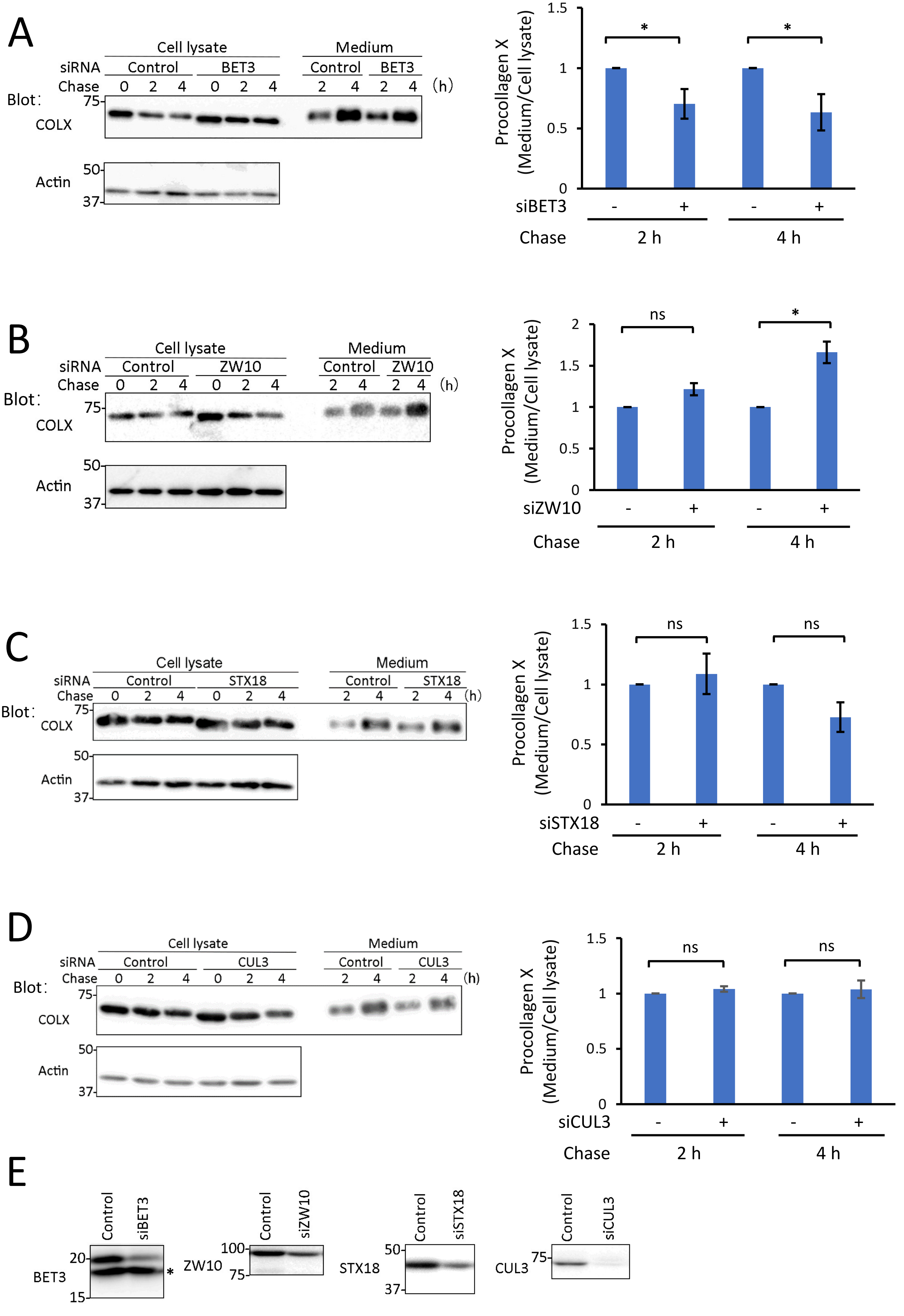
A
Cell lysate
Medium
siRNA Control BET3 Control BET3
Chase 0 2 4 0 2 4 2 4 2 4 (h)
Blot:
COLX
Actin
75
50
37
Procollagen X (Medium/Cell lysate)
siBET3 - + - +
Chase 2 h 4 h
*
B
siRNA Control ZW10 Control ZW10
Procollagen X (Medium/Cell lysate)
siZW10 - + - +
Chase 2 h 4 h
ns
*
C
siRNA Control STX18 Control STX18
siSTX18 - + - +
ns
D
siRNA Control CUL3 Control CUL3
siCUL3 - + - +
E
Control
siBET3
BET3
20
15
ZW10
siZW10
100
STX18
siSTX18
CUL3
siCUL3

Suppl. Fig. 7

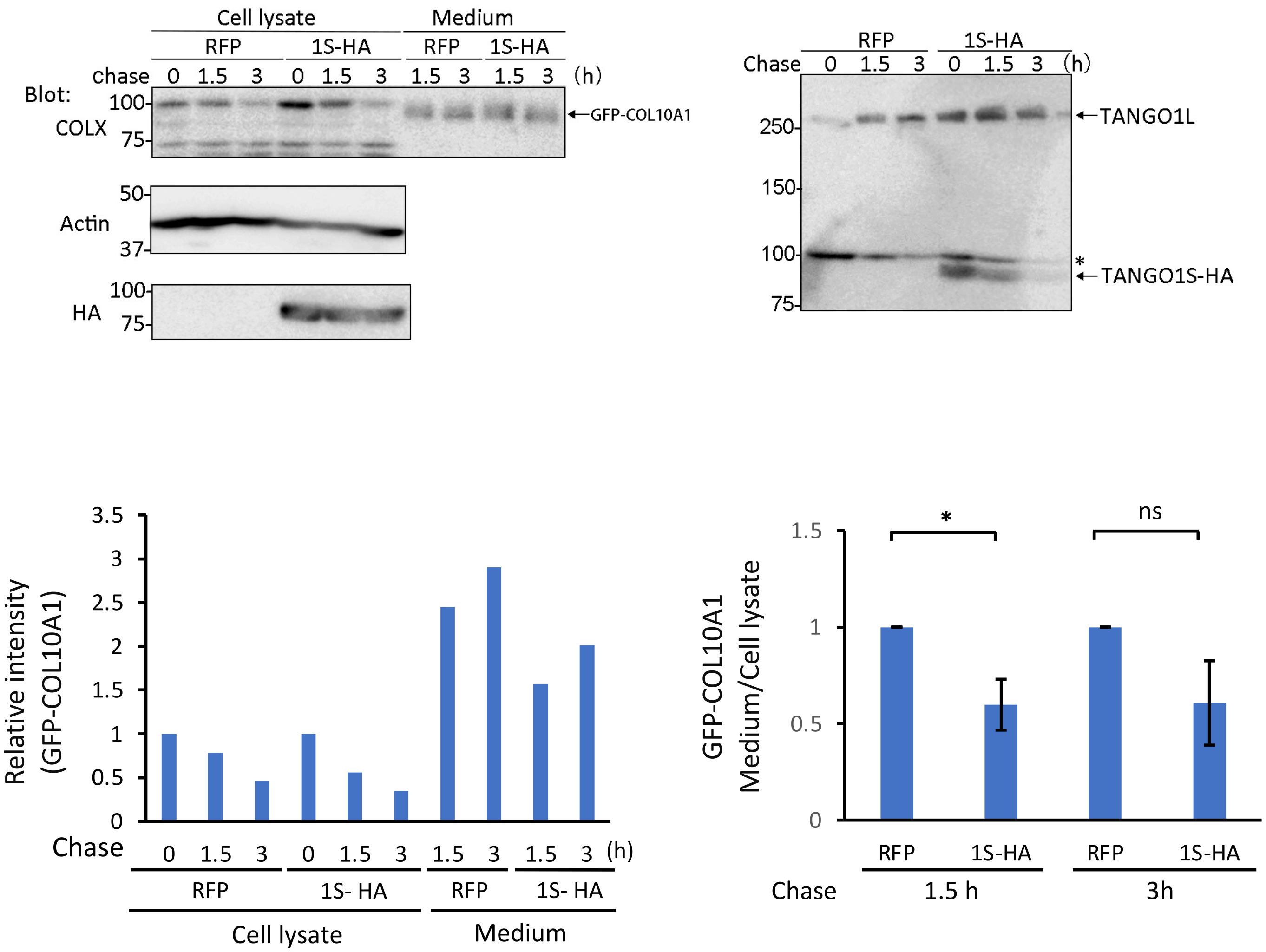

Cell lysate
Medium
RFP
1S-HA
RFP
1S-HA
chase 0 1.5 3 0 1.5 3 1.5 3 1.5 3 (h)
Blot:
COLX
100
75
GFP-COL10A1
Actin
50
37
HA
100
75
RFP
1S-HA
Chase 0 1.5 3 0 1.5 3 (h)
250
150
100
75
TANGO1L
*
TANGO1S-HA
Relative intensity (GFP-COL10A1)
3.5
3
2.5
2
1.5
1
0.5
0
Chase 0 1.5 3 0 1.5 3 1.5 3 1.5 3 (h)
RFP
1S- HA
RFP
1S- HA
Cell lysate
Medium
GFP-COL10A1 Medium/Cell lysate
1.5
1
0.5
0
*
ns
RFP
1S-HA
RFP
1S-HA
Chase 1.5 h
3h

Suppl. Fig. 8

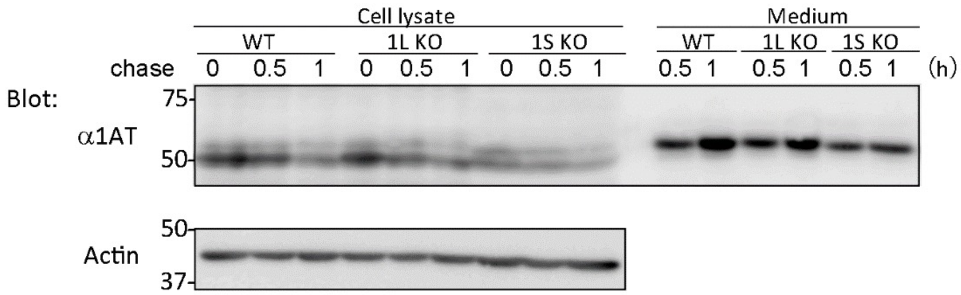

Cell lysate
Medium
WT
1L KO
1S KO
chase
0 0.5 1 0 0.5 1 0 0.5 1
0.5 1 0.5 1 0.5 1 (h)
Blot:
α1AT
75
50
Actin
50
37

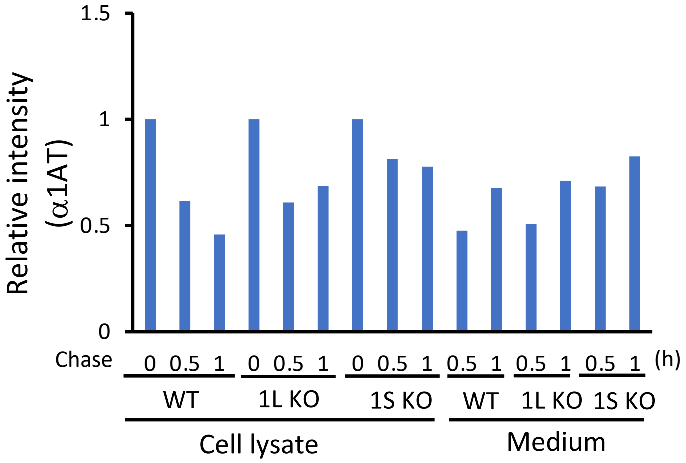

Relative intensity (α1AT)
1.5
1
0.5
0
Chase
0 0.5 1 0 0.5 1 0 0.5 1 0.5 1 0.5 1 0.5 1 (h)
WT 1L KO 1S KO WT 1L KO 1S KO
Cell lysate
Medium

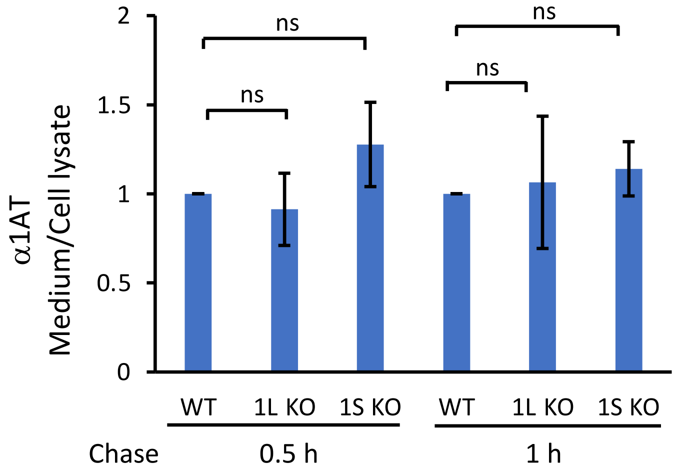

α1AT Medium/Cell lysate
2
1.5
1
0.5
0
ns
ns
ns
ns
WT 1L KO 1S KO WT 1L KO 1S KO
Chase
0.5 h
1 h

Suppl. Fig. 9

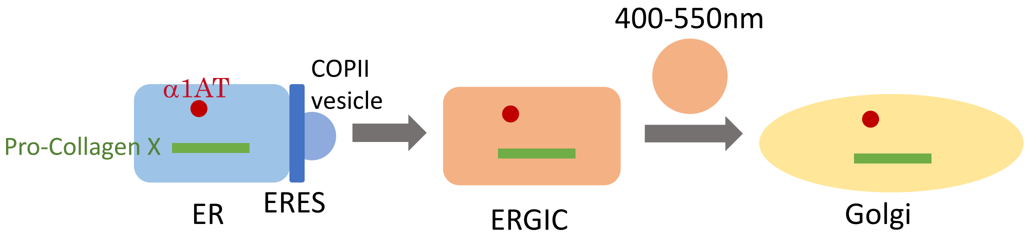
400-550nm
COPII
vesicle
α1AT
Pro-Collagen X
ER
ERES
ERGIC
Golgi
