# Supplementary material for: Visualization of ER-to-Golgi trafficking of procollagen X
Source: Cell Struct Funct. 2024 Sep 6;49(2):67–81. doi: 10.1247/csf.24024 (PMC11930776; doi:10.1247/csf.24024)
Supplement: Supplementary file 7 — Supplementary Materials [file csf_49_24024_7.zip › 49_24024_2.docx]

**Supplemental figure legends**

**Suppl. Fig. 1. Quantification of GFP-COL10A1, PDI-mCherry, and Golgi-BFP.**

1. Quantification of GFP-COL10A1 and PDI-mCherry in Fig. 1A. Relative fluorescence intensities on the indicated lines were analyzed using Leica LAS AF. Correlation diagram of GFP and mCherry signals are shown and Pearson correlation coefficient was calculated.
2. Quantification of GFP-COL10A1 and Golgi-BFP in Fig. 1B. Same as in Suppl. Fig. 1A, except GFP-COL10A1 and Golgi-BFP were quantified. Fluorescence intensities in Golgi and ER regions are also separately quantified.

**Suppl. Fig. 2. GFP-COL10A1 is secreted and deposited in the extracellular space.**

1. Live-cell imaging of HT-1080 and p52 cells transiently expressing GFP-COL10A1 (green). Cells were cultured in the presence of ascorbate for the indicated number of days. White arrows indicate GFP-COL10A1 deposited in the extracellular space. Cells are outlined by white dotted lines. Scale bar, 20 μm.
2. Immunostaining of p52 cells. Cells were fixed, either permeabilized or not permeabilized, and stained with anti-type X collagen (green) and anti-CRT (red) antibodies. White arrows indicate type X collagen deposited in the extracellular space. Cells are outlined by white dotted lines. Scale bars, 10 μm (upper panels) and 5 μm (lower panels).

**Suppl. Fig. 3. Transport of GFP-COL10A1-containing vesicles to the Golgi apparatus.**

Live-cell imaging of HT-1080 cells transiently expressing GFP-COL10A1 (green) and Golgi-BFP (blue) by confocal microscopy at ~40 min after addition of ascorbic acid. Time-lapse images were obtained every 1.5 sec for 3 min after photo-bleaching the Golgi area (lower panels, white dotted boxed region). Times denote the period after identification of a trafficking vesicle. Arrows indicate a GFP-COL10A1-containing vesicle trafficking from the ER to the Golgi. Scale bars, 5 μm (upper panels) and 2 μm (time-lapse).

**Suppl. Fig. 4. GFP-COL10A1 is transported by tubular structures that contain RAB1B.**

Same as in Suppl. Fig. 3, except HT-1080 cells transiently expressing GFP-COL10A1 (green) and mScarlet-RAB1B (red) were investigated. Scale bars, 5 μm (upper panels) and 2 μm (time-lapse).

**Suppl. Fig. 5. GFP-COL10A1-carrying vesicles contain ERGIC membranes.**

Same as in Suppl. Fig. 3, except HT-1080 cells transiently expressing GFP-COL10A1 (green) and mCherry-ERGIC53 (red) were investigated. Scale bars, 5 μm (upper panels) and 2 μm (time-lapse).

**Suppl. Fig. 6. BET3/TRAPPC3, but not ZW10, STX18, or CUL3, is required for ER-to-Golgi transport of procollagen X.**

Western blot analysis of p52 cells. Forty-eight hours after treatment with siRNA targeting BET3/TRAPPC3 **(A)**, ZW10 **(B)**, STX18 **(C)**, or CUL3 **(D)**, cells were cultured in SFM containing CHX and ascorbate, and were chased for the indicated periods. After TCA precipitation of the cell lysate and culture medium, procollagen X was analyzed by western blotting. Actin was used as a loading control. Procollagen X secreted into the medium was normalized to that in the cell lysate at chase 0 h, and the ratio versus cells treated with negative control siRNA is shown. Mean ± s.d. of three independent experiments. **P* < 0.05; ns, not significant (two-tailed Student’s *t*-test). Expression of BET3/TRAPPC3, ZW10, STX18, and CUL3 is shown in (**E**). The asterisk indicates a signal non-specifically detected by the anti-BET3/TRAPPC3 antibody.

**Suppl. Fig. 7. Transduction of TANGO1S-KO HeLa cells with TANGO1S-HA partly rescues the abnormal secretion of GFP-COL10A1.**

Western blot analysis of TANGO1S-KO cells. RFP or TANGO1S-HA was transduced by lentivirus, GFP-COL10A1 was transfected, and cells were incubated for a further 2 days. The cell lysate and culture medium were collected and analyzed as described in Suppl. Fig. 6. The asterisk indicates a signal non-specifically detected by the anti-TANGO1 antibody. Actin was used as a loading control. The relative intensity of GFP-COL10A1 was quantified (left graph), and the ratio of GFP-COL10A1 secreted into the medium was normalized to that in cells expressing RFP (right graph). Mean ± s.d. of three independent experiments. **P* < 0.05; ns, not significant (two-tailed Student’s *t*-test).

**Suppl. Fig. 8. Transport of α1AT in TANGO1L- and TANGO1S-KO HeLa cells.**

Western blot analysis of TANGO1L- and TANGO1S-KO cells. Same as in Suppl. Fig. 7, except α1AT was transduced by lentivirus into WT, TANGO1L-KO, and TANGO1S-KO HeLa cells. Cells were cultured in SFM containing CHX and chased for the indicated periods at 3 days after transduction. Cell lysates were added to Laemmli’s buffer, and the culture medium was collected and proteins were precipitated with TCA. The relative intensity of α1AT was quantified (left graph), and the ratio of α1AT secreted into the medium was normalized to that in WT HeLa cells (right graph). Mean ± s.d. of three independent experiments. ns, not significant (two-tailed Student’s *t*-test).

**Suppl. Fig. 9. Scheme of ER-to-Golgi transport of procollagen X.**

Procollagen X exits the ER in COPII vesicles at ERES, fuses to the ERGIC, and is then transported to the Golgi in vesicles with a diameter of 400–550 nm. These vesicles also contain conventional cargoes like α1AT, suggesting that procollagen X is secreted via the conventional transport pathway.

**Movie legends**

**Movie 1.** Live-cell imaging of HT-1080 cells transiently expressing GFP-COL10A1 (green) and PDI-mCherry (red). After addition of ascorbic acid, the Golgi region was photo-bleached. Time-lapse images were acquired every 1.5 sec using a confocal microscope (Leica SP8). The time point at which the vesicle was identified was set to 0.0 sec. Arrows indicate a GFP-COL10A1-containing vesicle trafficking from the ER to the Golgi. Individual time-lapse images are shown in Figure 2A. Scale bar, 2 μm.

**Movie 2.** Same as in Movie 1, except live-cell imaging of HT-1080 cells transiently expressing GFP-COL10A1 (green) and Golgi-BFP (blue) was performed. Arrows indicate a GFP-COL10A1-carrying vesicle moving from the ER to the Golgi. Individual time-lapse images are shown in Suppl. Fig. 3. Scale bars, 2 μm.

**Movie 3.** Same as in Movie 1, except live-cell imaging of HT-1080 cells transiently expressing GFP-COL10A1 (green) and mCherry-α1AT (red) was performed. Arrows indicate a vesicle carrying both GFP-COL10A1 and mCherry-α1AT moving from the ER to the Golgi. Individual time-lapse images are shown in Figure 3A. Scale bars, 2 μm.

**Movie 4.** Same as in Movie 1, except live-cell imaging of HT-1080 cells transiently expressing GFP-COL10A1 (green) and mScarlet-RAB1B (red) was performed. Arrows indicate a vesicle positive for both COL10A1 and mScarlet-RAB1B trafficking from the ER to the Golgi. Individual images are shown in Figure 4A. Scale bars, 2 μm.

**Movie 5.** Same as in Movie 4, except arrows indicate a tubular structure carrying both COL10A1 and mScarlet-RAB1B trafficking from the ER to the Golgi. Individual images are shown in Suppl. Fig. 4. Scale bars, 2 μm.

**Movie 6.** Same as in Movie 1, except live-cell imaging of HT-1080 cells transiently expressing GFP-COL10A1 (green) and mCherry-ERGIC53 (red) was performed. Arrows indicate a vesicle positive for both COL10A1 and mCherry-ERGIC53 moving from the ER to the Golgi. Individual images are shown in Suppl. Fig. 5. Scale bars, 2 μm.
